# A complex of Neuroplastin and Plasma Membrane Ca^2+^ ATPase controls T cell activation

**DOI:** 10.1038/s41598-017-08519-4

**Published:** 2017-08-21

**Authors:** Mark Korthals, Kristina Langnaese, Karl-Heinz Smalla, Thilo Kähne, Rodrigo Herrera-Molina, Juliane Handschuh, Anne-Christin Lehmann, Dejan Mamula, Michael Naumann, Constanze Seidenbecher, Werner Zuschratter, Kerry Tedford, Eckart D. Gundelfinger, Dirk Montag, Klaus-Dieter Fischer, Ulrich Thomas

**Affiliations:** 1 Institute of Biochemistry and Cell Biology, Otto-von-Guericke-University, Medical Faculty, D-39120 Magdeburg, Germany; 2 Department of Neurochemistry and Molecular Biology, Leibniz Institute for Neurobiology, D-39118 Magdeburg, Germany; 3 Institute of Experimental Internal Medicine, Otto-von-Guericke-University, Medical Faculty, D-39120 Magdeburg, Germany; 4 Special Lab Electron and Laserscanning Microscopy, Leibniz Institute for Neurobiology, D-39118 Magdeburg, Germany; 5 Center for Behavioral Brain Sciences, D-39120 Magdeburg, Germany; 6 Medical Faculty, Otto von Guericke University, D-39120 Magdeburg, Germany; 7 German Center for Neurodegenerative Diseases (DZNE) Site Magdeburg, D-39120 Magdeburg, Germany; 8 Neurogenetics Special Laboratory, Leibniz Institute for Neurobiology, D-39118 Magdeburg, Germany

## Abstract

The outcome of T cell activation is determined by mechanisms that balance Ca^2+^ influx and clearance. Here we report that murine CD4 T cells lacking Neuroplastin (*Nptn* ^−/−^), an immunoglobulin superfamily protein, display elevated cytosolic Ca^2+^ and impaired post-stimulation Ca^2+^ clearance, along with increased nuclear levels of NFAT transcription factor and enhanced T cell receptor-induced cytokine production. On the molecular level, we identified plasma membrane Ca^2+^ ATPases (PMCAs) as the main interaction partners of Neuroplastin. PMCA levels were reduced by over 70% in *Nptn* ^−/−^ T cells, suggesting an explanation for altered Ca^2+^ handling. Supporting this, Ca^2+^ extrusion was impaired while Ca^2+^ levels in internal stores were increased. T cells heterozygous for PMCA1 mimicked the phenotype of *Nptn* ^−/−^ T cells. Consistent with sustained Ca^2+^ levels, differentiation of *Nptn* ^−/−^ T helper cells was biased towards the Th1 versus Th2 subset. Our study thus establishes Neuroplastin-PMCA modules as important regulators of T cell activation.

## Introduction

Activation of the T cell receptor (TCR) by cognate peptide on an antigen-presenting cell (APC) leads to release of Ca^2+^ from the endoplasmic reticulum (ER), followed by store-operated Ca^2+^ entry (SOCE)^1, 2^. Cytosolic Ca^2+^ levels ([Ca^2+^]_i_) return to baseline due to both Ca^2+^ uptake into stores and extrusion across the plasma membrane *via* PMCAs^3^. The degree to which the TCR is engaged by ligands on the APC together with extrinsic factors, such as cytokines, determines the amplitude and the spatio-temporal profile of Ca^2+^ signals^4^. The Ca^2+^ signal acts on calcineurin, a phosphatase, which in turn dephosphorylates nuclear factor of activated T cells (NFAT), triggering its nuclear translocation and thus transcription of T cell activation genes^4–6^. Products of these genes include cytokines that induce polarization of T cells to T helper 1 (Th1) or T helper 2 (Th2) profiles to direct cellular or humoral responses, respectively^7^. Th1 differentiation strongly correlates with sustained TCR-induced Ca^2+^ signaling, while the Th2 profile is associated with weaker and more transient Ca^2+^ signals^8–10^.

The PMCA family comprises four members, two of which are expressed in T cells: PMCA1 and PMCA4^11–13^. Loss of PMCA1 in mice is embryonic lethal, while PMCA4 knock out mice are viable but male-sterile^11^. PMCAs are controlled at the transcriptional, splicing and post-translational level^13, 14^. In Jurkat T cells, PMCA4 activity is stimulated by Ca^2+^ ^3^ but inhibited through spatial confinement in microdomains at the immune synapse, where Ca^2+^ sequestration by mitochondria or association with membrane proteins STIM1 and POST restricts its local activity at the synapse^15–17^. These modifications to PMCA localization ensure that TCR-induced Ca^2+^ signaling succeeds in activating downstream targets such as NFAT.

Neuroplastin is a transmembrane protein of the Ig superfamily and a close paralog of CD147 (Basigin/EMMPRIN)^18^. CD147 is involved in T cell development and activation^19, 20^. In contrast, nothing is known about Neuroplastin in T cells. Neuroplastin is expressed as two highly gylcosylated splice variants called Np65 and Np55, with 3 and 2 Ig domains, respectively^18, 21^. Np65 exhibits trans-homophilic binding^22^ and is mainly expressed in neurons, while Np55 is more broadly expressed^21^. Np65 is required for learning and memory^23^ and for synaptic structure and plasticity^22, 24, 25^. To date, few non-neuron-specific interaction partners of Np55 have been identified^26–28^. We report here that Neuroplastin interacts with PMCA and is required for stabilizing PMCA expression. *Nptn* ^−/−^ T cells show elevated Ca^2+^ levels and increased nuclear NFAT, and this phenotype was mimicked in PMCA1 heterozygous T cells. Finally, we show that loss of Neuroplastin from T cells results in the production of elevated levels of cytokines and a strong bias towards Th1 polarization.

## Results

### Expression of Neuroplastin isoform Np55 in T cells

**Figure 1:**
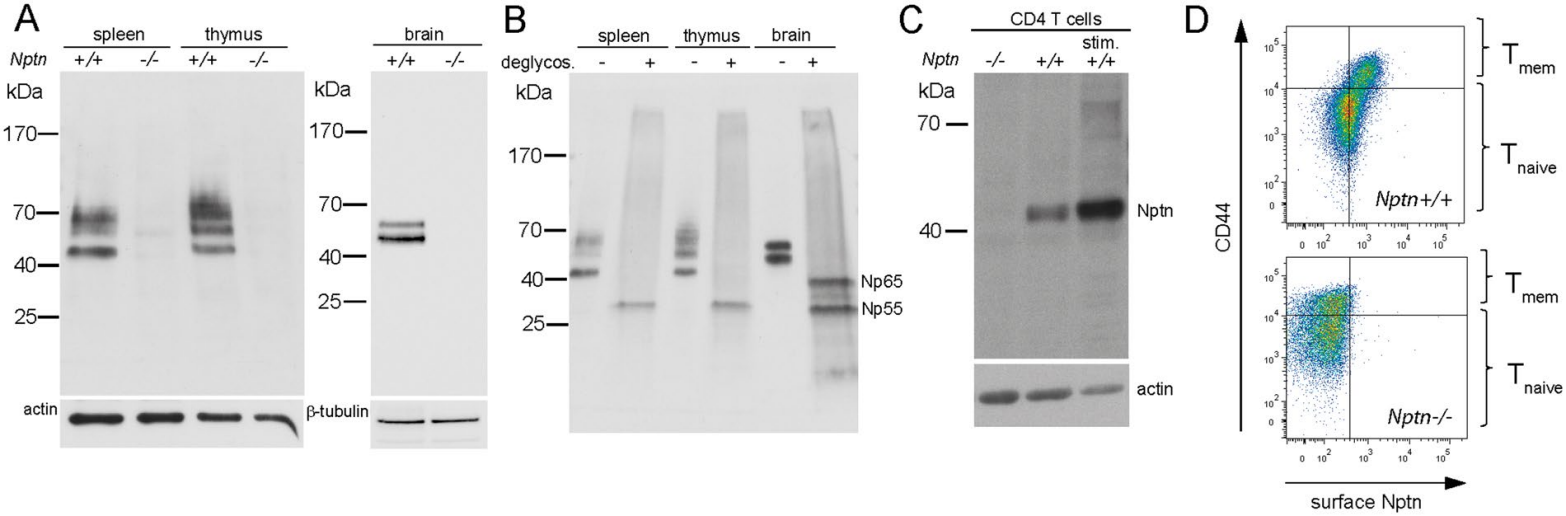
Expression of Neuroplastin in T cells. (**A**) Western blot detection of Neuroplastin in spleen- and thymus-derived Triton X-100 solubilized membrane fractions (30 μg/ lane) and brain control samples (10 µg) from *Nptn* ^+*/*+^ and *Nptn* ^−/−^ mice. (**B**) Western blot analysis of Neuroplastin before and after deglycosylation detected in spleen and thymus samples (15 µg each) and brain control samples. Bands of ~28 and ~40 kDa represent non-glycosylated Np55 and Np65. (**C**) Western blot detection of Neuroplastin (Nptn) in naïve and activated T cells reveals an apparent molecular weight of ~45 kDa and strong upregulation upon stimulation by culturing on immobilized anti-CD3/anti-CD28. (**D**) FACS analysis of Neuroplastin surface expression. Plots show Neuroplastin expression levels on CD44^−^ naïve and CD44^+^ memory CD4 T cells.

To determine if Neuroplastin isoforms are expressed in lymphocytes, we tested spleen and thymus extracts with an antibody directed against the common extracellular portion of both Np55 and Np65. We detected several specific bands that collapsed following deglycosylation to a single band of ~28 kDa, the calculated peptide mass of Np55, whereas two bands in brain extracts corresponded to Np55 and Np65 (Fig. 1A and B). Thus, as in other non-neuronal tissues^21^, differential glycosylation of Np55 accounts for the appearance of multiple bands in lymphoid tissues. In extracts from purified CD4 T cells, Neuroplastin was present as a band of 45 kDa that was upregulated in activated CD4 blast cells (Fig. 1C). Neuroplastin was surface-expressed on both CD44^low^ naïve and CD44^high^ memory T cells and was increased on the latter (Fig. 1D). To determine if Neuroplastin is required for T cell development, we reconstituted the immune system of irradiated wildtype (*wt*) mice with 1:1 mixtures of bone marrow cells derived from *wt* and *Nptn* ^−/−^ donors carrying specific cell surface markers (Ly5.1 *versus* Ly5.2). When competing with *wt* cells, *Nptn* ^−/−^ precursor cells within an otherwise normal cellular environment were moderately hindered in colonizing the thymus. However, progression through thymic developmental stages and peripheral T cell homeostasis appeared normal when assessed for relative abundance of *Nptn* ^−/−^ thymocyte and T cell subpopulations (Supplementary Fig. S1). Together, these data show that Np55 is expressed in T cells and upregulated upon activation, but is not essential for T cell development.

### Neuroplastin controls [Ca^2+^]_i_ and cytokine production

**Figure 2:**
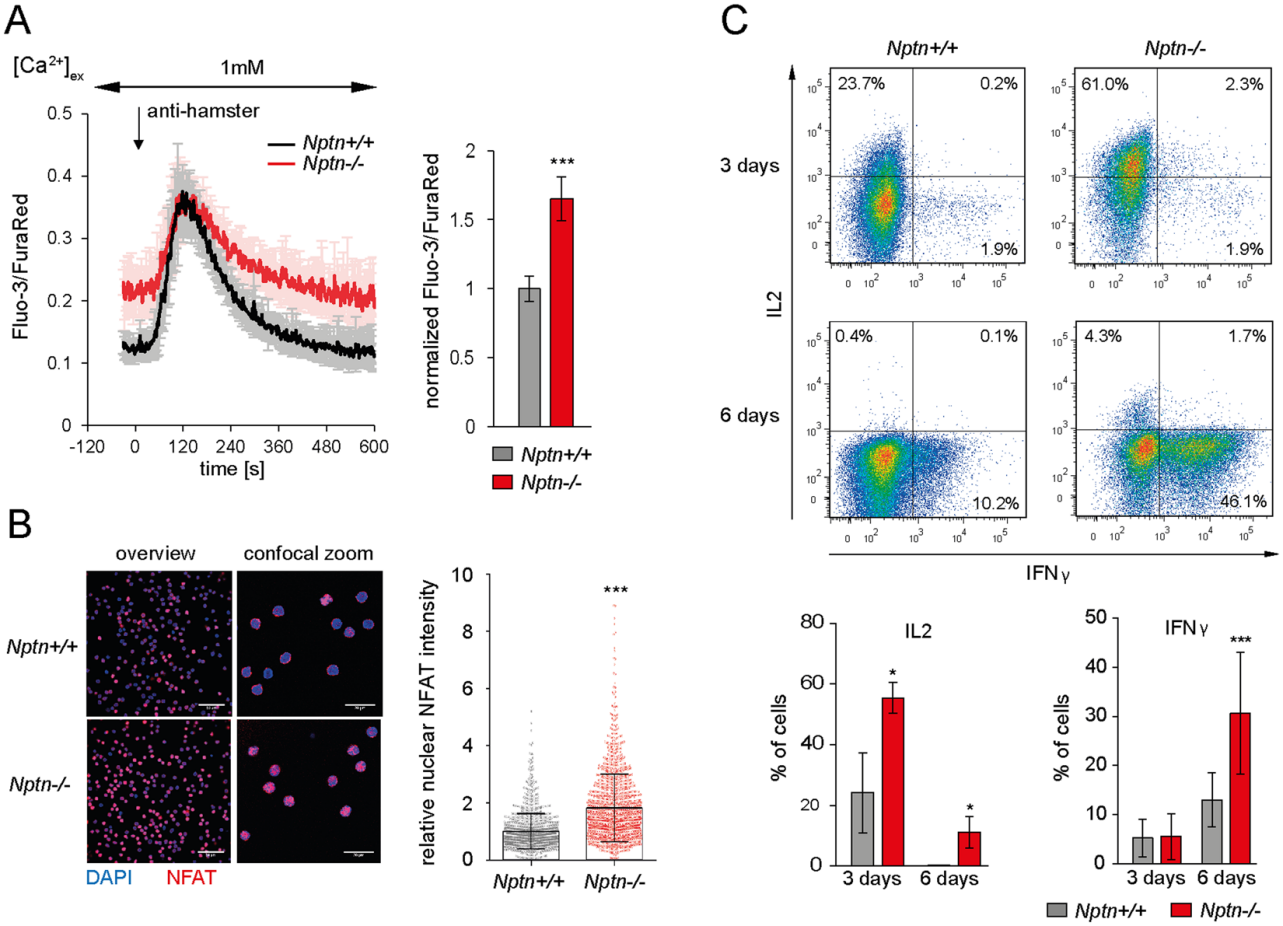
Increased Ca^2+^ levels and Ca^2+^-dependent signaling in *Nptn* ^−/−^ T cells. (**A**) Flow cytometric ratiometric Ca^2+^ measurements in T cells. Anti-CD3 labeled CD4 T cells in buffer containing 1 mM Ca^2^ were stimulated by crosslinking CD3 (anti-hamster). The diagram shows kinetics of the mean instantaneous single cell ratio ± SD of *wt* and *Nptn* ^−/−^ naive CD4 T cells from 3 experiments. The bar graph shows the mean normalized baseline ratios ± combined SD from 5 experiments, ***p < 0.001, unpaired two-tailed t-test. Further quantifications of baseline and peak levels and the decay phase are summarized in Supplementary Table S1. (**B**) Confocal imaging of nuclear NFAT localization. Images show NFAT immunofluorescence and DAPI staining in *wt* and *Nptn* ^−/−^ T cells at different magnifications from separate samples. Scale bars: left, 50 μm; right, 20 μm. Normalized nuclear NFAT intensities of 1299 *wt* and 1456 *Nptn* ^−/−^ single cells from 5 experiments are presented, overlay bars showing mean normalized values ± combined SD from these 5 experiments, ***p < 0.001, unpaired two-tailed t-test. (**C**) Detection of cytokines in stimulated *wt* and *Nptn* ^−/−^ T cells by intracellular FACS. Representative FACS plots show IL2 and IFNγ expression in CD4 T cells cultured for 3 or 6 days on immobilized anti-CD3. Mean proportions of IL2 and IFNγ producing cells ± SD derived from 3 and 8 experiments, respectively, are shown. *p < 0.05, ***p < 0.001, unpaired two-tailed t-test.

We next analyzed TCR-proximal signaling in *Nptn* ^−/−^ T cells. TCR-induced cell proliferation, CD69 upregulation, and activation of the protein kinase ERK1/2 were normal (Supplementary Fig. S1). TCR-induced Ca^2+^ flux showed normal peak levels, however, basal [Ca^2+^]_i_ was elevated by ~65% in *Nptn* ^−/−^ T cells (Fig. 2A and Supplementary Table S1). To determine if increased basal [Ca^2+^]_i_ level impacted downstream signaling, we tested nuclear levels of NFAT. Nuclear NFATc2 was strongly increased in *Nptn* ^−/−^ *ex vivo* T cells (Fig. 2B). To verify that these defects were intrinsic to the T cells, we also analyzed mixed bone marrow chimeras and found that the *Nptn* ^−/−^ but not *wt* T cells showed the same phenotype. Of note, compared to *wt* T cells a much larger portion of the naïve *Nptn* ^−/−^ T cells showed strongly increased instantaneous basal [Ca^2+^]_i_ in single cells relative to the mean value (Supplementary Fig. S2).

**Figure 3:**
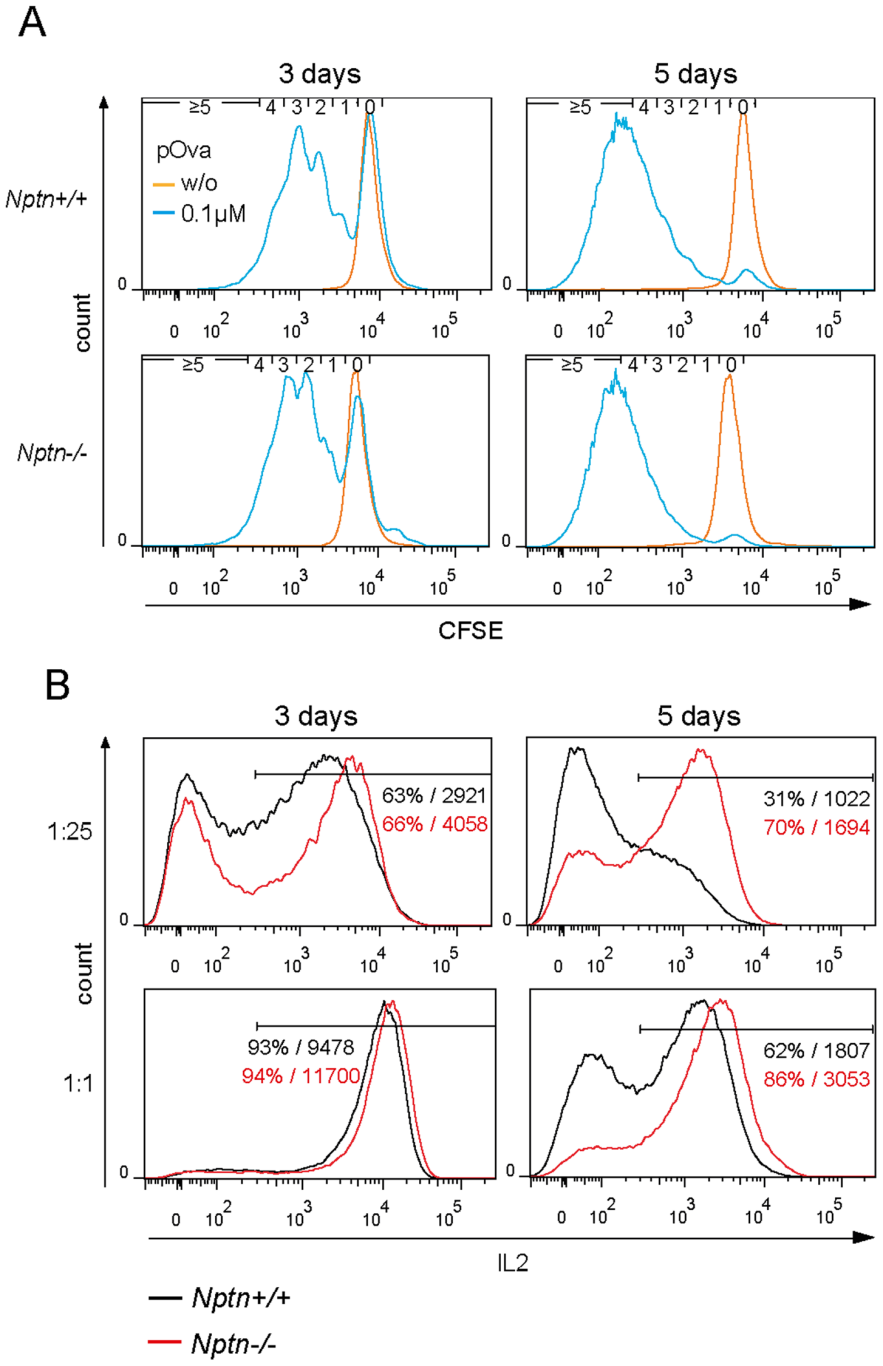
Antigen-induced IL2 production is facilitated in *Nptn* ^−/−^ T cells. (**A**) Proliferation upon antigen-specific T cell stimulation. 1 × 10^5^ OT-II transgenic *wt* or *Nptn* ^−/−^ CD4 T cells were cocultured for 3 or 5 days together with 4 × 10^3^ (1:25) dendritic cells (DCs) which had been loaded either with 0.1 µM pOva or were left unloaded. Proliferation of T cells was analyzed flow cytometrically by CFSE dilution. T cells and DCs were distinguished based on CD4 and CD11b labeling, respectively. Histograms represent results from one of two independent experiments. Small numbers indicate number of completed cell cycles. (**B**) Production of IL2 by OT-II transgenic T cells cocultured with DCs as described above with DC: T cell ratios of 1:25 or 1:1, respectively, was measured by intracellular FACS. Representative histograms show IL2 fluorescence intensities from one of two experiments. Numbers indicate proportions of IL2 producing T cells and mean fluorescence intensity of IL2 within the IL2 positive population.

Nuclear NFAT is required for transcription of pro-inflammatory cytokines^6^. We therefore tested TCR-induced production of IL2 and IFNγ in *Nptn* ^−/−^ CD4 T cells *ex vivo*. We observed significant rises in IL2 after 3 and 6 days and in IFNγ after 6 days of TCR stimulation (Fig. 2C). Additionally, we stimulated CD4 T cells carrying an OT-II TCR transgene with dendritic cells (DC) presenting the cognate OVA peptide. We found that proliferation of *Nptn* ^-/−^ cells was normal (Fig. 3A), similar to the T cells stimulated by the TCR (Supplementary Fig. S1). With a DC to T cell ratio of 1:1, IL2 production by *Nptn* ^−/−^ cells was increased as compared to *wt* cells (Fig. 3B). This difference was much more pronounced at a ratio of 1:25 DC to T cells. Thus the activation threshold for cytokine production is lowered in *Nptn* ^−/−^ T cells. Together, these results show that loss of Neuroplastin in T cells results in elevated basal Ca^2+^ and leads to increased production of NFAT-regulated cytokines.

### Mass spectrometric analysis reveals PMCAs as Neuroplastin-binding partners

**Figure 4:**
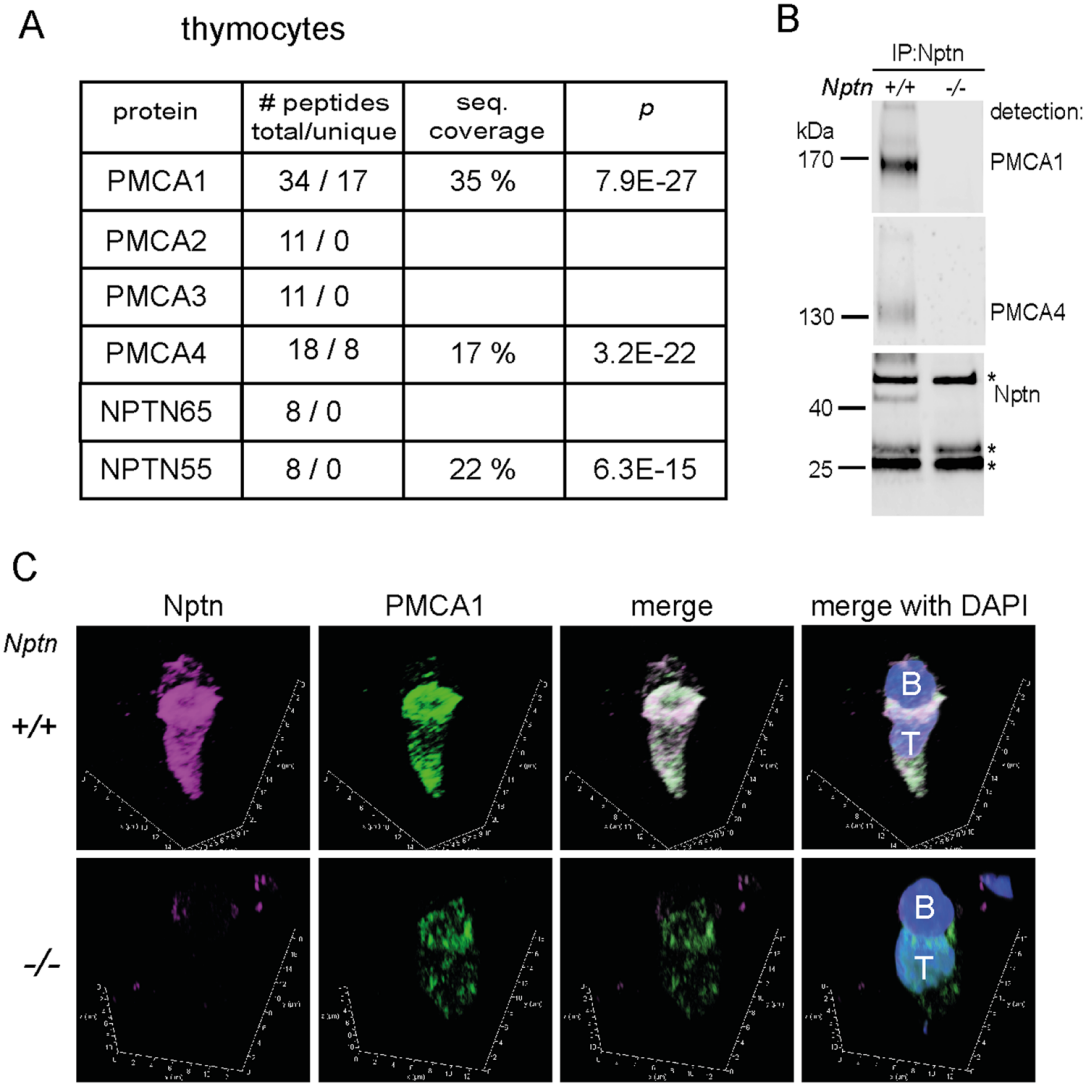
PMCA1 and PMCA4 are Neuroplastin-binding partners. (**A**) Table showing Neuroplastin interaction with PMCA1 and 4 in thymocytes revealed by MS of Neuroplastin-immunoprecipitates (IP) from *wt* thymocyte solubilizates. No unique peptides were detected for PMCA2 or 3. (**B**) Verification of the Neuroplastin-PMCA complex by Western blot detection of PMCA1 and 4 in Neuroplastin-IP. Asterisks mark immunoglobulin light and heavy chain bands. *Nptn* ^−/−^ control samples confirmed specificity. (**C**) Immunofluorescent double labeling of Neuroplastin (magenta) and PMCA1 (green) and co-staining for DNA (DAPI, blue) on conjugates formed between pOva-loaded B cells and OT-II transgenic *wt* (upper panel) or *Nptn* ^−/−^ (lower panel) CD4 T cell blasts, scanned at 63x magnification and shown as 3D reconstructions from confocal stacks. Merged images reveal striking co-localization (white) of both proteins at the synaptic interface between the elongated *wt* T cell (T) and the weakly labeled B cell (B). Note that reduced PMCA1 in *Nptn* ^−/−^ T cells still enriches at the immune synapse.

To gain insight into how Neuroplastin controls Ca^2+^ levels in T cells, we sought to identify Neuroplastin-interacting proteins. We therefore used liquid chromatography-mass spectrometry to interrogate Neuroplastin-immunoprecipitates from *wt* and *Nptn* ^−/−^ thymocytes. The two proteins with the highest score and with an obvious link to Ca^2+^ homeostasis were PMCA1 and PMCA4 (Fig. 4A and Supplementary Fig. S3), both represented by unique peptides (Supplementary Fig. S3). The absence of peptides specific for PMCA2 and PMCA3 is consistent with very low transcript levels for these isoforms in immune cells as deduced from database entries (www.immgen.org ^12^). Western blot analysis confirmed that Neuroplastin-immunoprecipitates from *wt* thymic lysates contained PMCA1 and PMCA4 (Fig. 4B).

We next elaborated on the interaction between PMCAs and Neuroplastin by microscopy. To our knowledge, reports on the subcellular distribution of endogenous PMCAs in T cells are lacking to date. Previous studies, however, have shown that EGFP-tagged PMCA4 enriches at immune synapses when expressed in Jurkat T cells^16, 17^. We found that expression of Np55-EGFP in Jurkat cells also resulted in its localization to immune synapses, peripheral to the TCR (Supplementary Fig. S4). To assess the extent of co-localization of endogenous Neuroplastin and PMCA1 in primary T cells, we performed immunofluorescent double labelings on conjugates between *wt* OVA peptide-loaded B cells and OT-II transgenic *wt* or *Nptn* ^−/−^ CD4 T cells, respectively. Confocal microscopy revealed strong co-localization of immunofluorescent signals for Neuroplastin and PMCA1, including co-enrichment at the immune synapse in *wt* T cells (Fig. 4C). As expected, immunofluorescence for Neuroplastin was virtually absent in *Nptn* ^−/−^ T cells. Moreover, consistent with results presented below, we found that immunofluorescence for PMCA1 was diminished in *Nptn* ^−/−^ T cells. Of note, the remaining PMCA1 still displayed synaptic enrichment. Together, these findings show that Neuroplastin associates with PMCA in T cells and that both proteins co-enrich at the immune synapse.

### Neuroplastin controls PMCA levels

**Figure 5:**
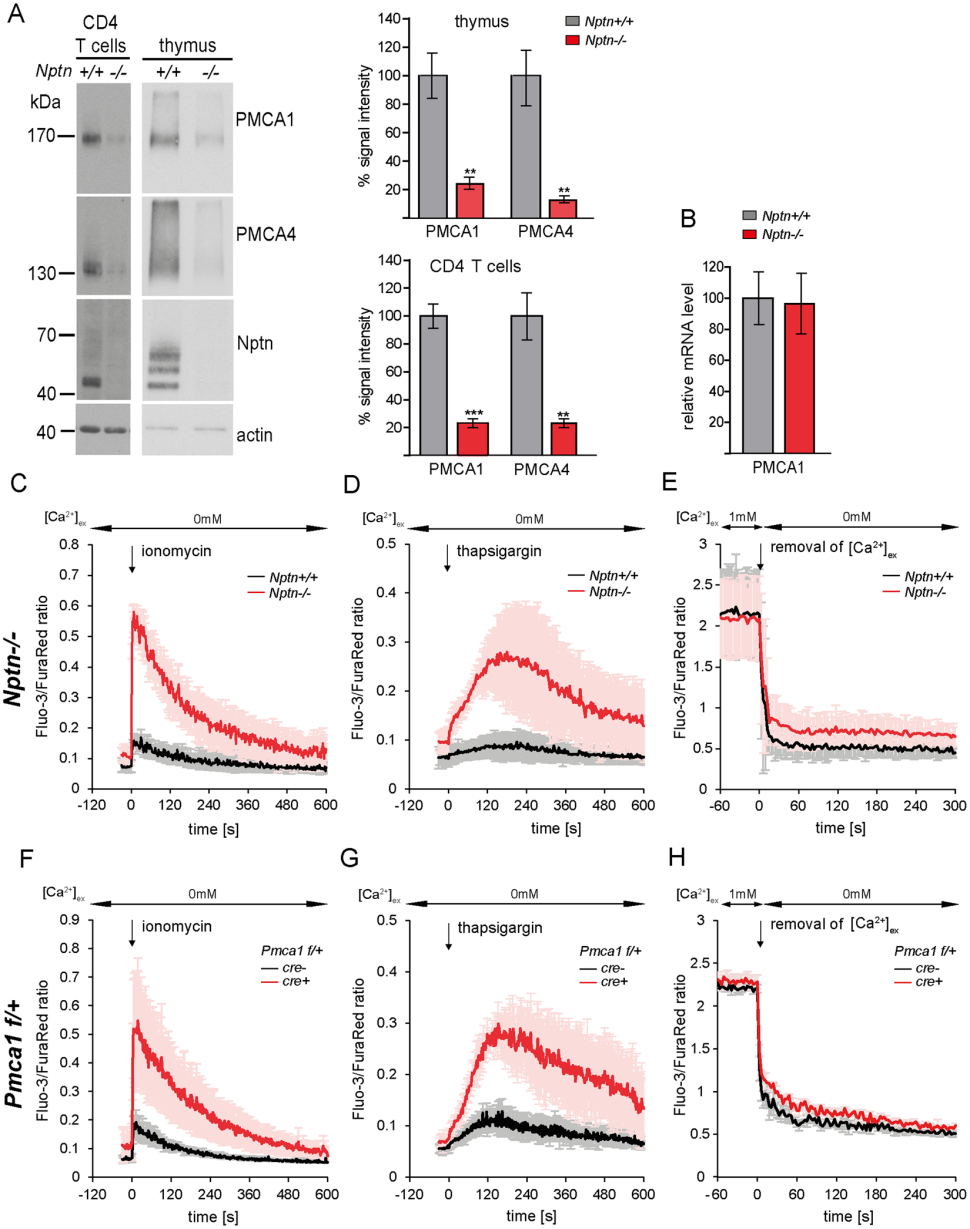
Neuroplastin is required for PMCA stabilization and Ca^2+^ clearance. (**A**) Western blot analysis of PMCA1, PMCA4 and Neuroplastin in *wt* and *Nptn* ^−/−^ samples from CD4 T cells and thymus. Representative blots show lack of Neuroplastin and strong reduction of PMCA1 and PMCA4 in *Nptn* ^−/−^ CD4 T cells and thymus. Signal intensities were quantified by densitometry using β-actin signals for normalization. Bar graphs show mean ± SEM from 4 *wt* and 4 *Nptn* ^−/−^ replicate thymus samples, and from 3 samples of CD4 T cell extracts each, **p < 0.01, ***p < 0.001, unpaired two-tailed t-test. (**B**) qRT-PCR of PMCA1 transcript levels in lymph node cells. *B2m* and *Hprt* served as references for normalization. Graph shows mean relative transcript levels ± SD derived from 7 samples per group. (**C**–**E**) Flow cytometric ratiometric measurement of post-stimulatory Ca^2+^ clearance in *wt* or *Nptn* ^−/−^ naïve CD4 T cells. After baseline recording, Ca^2+^ release from internal stores was induced in Ca^2+^-free buffer by 1 μg/ml ionomycin. Kinetics of the mean Fluo-3/FuraRed ratio ± SD from 3 experiments is shown (**C**). Ca^2+^ release from the ER was induced by 1 μg/ml thapsigargin in Ca^2+^ -free buffer. Mean ratios ± SD from 4 experiments are shown (**D**). Ca^2+^ clearance across the plasma membrane, investigated after thapsigargin treatment as in (**D**) and consecutive induction of SOCE by resuspending cells in buffer containing 1 mM Ca^2+^. High Fluo-3/FuraRed ratios were recorded for 1 min before Ca^2+^ clearance was induced by resuspending cells in Ca^2+^ -free buffer again, and recording was immediately continued. Mean ratios ± SD from 5 experiments are shown (**E**). (**F**,**G**) Ca^2+^ levels in *Pmca1* haploinsufficient T cells. Ca^2+^ release from internal stores induced by ionomycin (F) or thapsigargin (**G**) were repeated exactly as in (**C**) and (**D**), respectively, but with T cells from *Pmca1* ^*f/*+^ mice expressing either a T cell-specific Cre recombinase (*cre*+) or no Cre (*cre*−). Mean Fluo-3/FuraRed ratios ± SD were derived from 3 experiments each. Quantifications of the Ca^2+^ decay shown in (**E**) and (**H**) by fitting exponential curves are summarized in Supplementary Tables 1 and 2, respectively.

While testing Neuroplastin-immunoprecipitates, we consistently observed reduced expression of PMCA1 and PMCA4 in input controls from *Nptn* ^−/−^ cells. Quantitative immunoblot analyses revealed that both isoforms were reduced by over 75% in *Nptn* ^−/−^ samples from thymi and CD4 T cells compared to *wt* controls (Fig. 5A). qRT-PCR for PMCA1 from *wt* and *Nptn* ^−/−^ lymphocytes revealed no differences in transcript levels (Fig. 5B), showing that loss of PMCA was post-transcriptional.

To test if PMCA and Neuroplastin associate at the cell surface, we aimed to immunoprecipitate Neuroplastin from purified surface proteins, however, a limitation in using primary T cells is the difficulty in obtaining enough membrane material. Therefore, we used bone marrow-derived macrophages (BMDM). We first confirmed that Neuroplastin interacts with PMCA1 and PMCA4 in BMDMs by co-immunoprecipitation (Supplementary Fig. S5). Both isoforms were greatly reduced in the absence of Neuroplastin (Supplementary Fig. S5). As in T cells, the mRNA of PMCA1 was normal in *Nptn* ^−/−^ BMDM, indicating that Neuroplastin stabilizes PMCA at the protein level (Supplementary Fig. S5). We then biotinylated cell surface proteins and affinity-purified them. PMCA1 was detectable in resulting eluates but not in non-biotinylated control samples (Supplementary Fig. S5). Notably, despite the expected reduction of PMCA1 in the *Nptn* ^−/−^ samples, the presence of biotinylated PMCA showed that it could still reach the cell surface (Supplementary Fig. S5). We then immunoprecipitated Neuroplastin complexes from biotinylated cells, affinity purified the surface biotinylated portion of the immunoprecipitate, and probed for Neuroplastin and PMCA1. As expected, PMCA1 was associated with surface Neuroplastin, demonstrating that the two proteins interact at the cell membrane (Supplementary Fig. S5).

We next sought to identify the subcellular compartment where Neuroplastin is required for PMCA stabilization. To this end, we used an Optiprep^TM^ gradient to fractionate *wt* and *Nptn* ^−/−^ BMDMs by membrane compartments identified by specific markers, and probed for PMCA (Supplementary Fig. S5). Compared to *wt*, PMCA1 immunoreactivity in *Nptn* ^−/−^ samples was evenly reduced in all compartments assessed, including plasma, Golgi and ER membranes (Supplementary Fig. S5). Collectively, these results suggest that Neuroplastin stabilizes PMCA already at an early stage of biosynthesis and that they interact throughout the secretory pathway up to the cell surface.

### Neuroplastin and PMCA control T cell Ca^2+^ clearance

The reduction of PMCA in the absence of Neuroplastin suggested an explanation for the increased basal Ca^2+^ in *Nptn* ^−/−^ T cells (Fig. 2A). Following TCR stimulation of CD4 T cells, the decay phase showed differences in Ca^2+^ clearance that were consistent with a defect in Ca^2+^ extrusion (Fig. 2A). Specifically, compared to *wt* T cells, the calculated initial rate of Ca^2+^ clearance (*Nptn* ^−/−^: 0.0016 a.u./s *vs wt*: 0.0029 a.u./s) and the rate constant K (0.0078 ± 0.0006 s^−1^ *vs* 0.0092 ± 0.0003 s^−1^) as determined by exponential fit were reduced in mutant T cells, whereas the plateau was elevated (0.206 ± 0.004 a.u. *vs* 0.115 ± 0.002 a.u.) (Supplementary Table S1). Still, given the strong reduction of both PMCAs, this phenotype appeared to be relatively mild. We therefore considered the possibility that stronger phenotypes were prevented by an increased uptake of Ca^2+^ into internal stores such as mitochondria or ER. To test this, we stimulated CD4 T cells in Ca^2+^-free medium with the Ca^2+^ ionophore ionomycin, and observed a rise in cytoplasmic Ca^2+^ mobilization that was about 5 times higher in *Nptn* ^−/−^ T cells than in *wt* (Fig. 5C). We then depleted ER Ca^2+^ with the SERCA inhibitor thapsigargin, which also led to a 5-fold higher increase of [Ca^2+^]_i_ in *Nptn* ^−/−^ T cells (Fig. 5D). To measure Ca^2+^ clearance following SOCE, we used an established protocol consisting of depletion of ER Ca^2+^ by thapsigargin, followed by the addition of extracellular Ca^2+^ ^29^. The resulting SOCE peak levels and the quick decline to a high level plateau were indistinguishable between *wt* and *Nptn* ^−/−^ T cells (Supplementary Fig. S6). Following this, removal of external Ca^2+^ triggered a steep decline of intracellular Ca^2+^. This phase of Ca^2+^ clearance was impaired in *Nptn* ^−/−^ T cells (Fig. 5E), again reflected by reductions in the initial clearance rate (0.388 a.u./s *vs* 0.522 a.u./s) and rate constant (0.306 ± 0.038 s^−1^ vs 0.354 ± 0.026 s^−1^) and by an elevated plateau (0.835 ± 0.026 a.u. *vs* 0.603 ± 0.016 a.u.) (Supplementary Table S1).

PMCAs are strongly reduced though not absent in *Nptn* ^−/−^ T cells. Therefore, to directly test whether a partial reduction of PMCA1 can cause phenotypes similar to those observed in *Nptn* ^−/−^ cells, we analyzed CD4 T cells from mice with a T cell-specific knock out of one allele of *Pmca1* (*pTα* ^*iCre*^ *Pmca1* ^*flox/*+^). First we observed that, similar to Neuroplastin, PMCA1 and PMCA4 were upregulated following T cell activation (Supplementary Fig. S7). Loss of one *Pmca1* allele resulted in a 40% reduction of PMCA1 protein (Supplementary Fig. S7). TCR stimulation of Ca^2+^ flux in *Pmca1* heterozygous CD4 T cells resulted in a normal peak of Ca^2+^ but revealed increased basal [Ca^2+^]_i_ and decreased Ca^2+^ clearance after stimulation (Supplementary Fig. S7 and Supplementary Table S2). Similarly, although less pronounced than for naïve *Nptn* ^−/−^ T cells (Supplementary Fig. S2), a larger fraction of the *Pmca1* ^+*/*−^ T cells showed much higher baseline [Ca^2+^]_i_ (Supplementary Fig. S7).

Notably, ionomycin and thapsigargin treatments confirmed that intracellular compartments store an excess of intracellular Ca^2+^ (Fig. 5F and G), similar to the *Nptn* ^−/−^ T cells. Ca^2+^ removal after SOCE also revealed a defect in Ca^2+^ clearance; although it was somewhat less severe than in the *Nptn* ^−/−^ T cells, possibly because there was more PMCA1 and PMCA4 present (Fig. 5H and Supplementary Table S2). Furthermore, *Pmca1* haploinsufficiency caused an increase in nuclear NFAT and TCR-induced cytokine production, again similar to *Nptn* ^−/−^ CD4 T cells (Supplementary Fig. S7). Based on these results, we conclude that the disrupted association of Neuroplastin with PMCA is solely responsible for the deregulation of Ca^2+^ homeostasis observed in *Nptn*-deficient CD4 T cells.

### Polarization of *Nptn*^−/−^ T cells favors the Th1 profile

**Figure 6:**
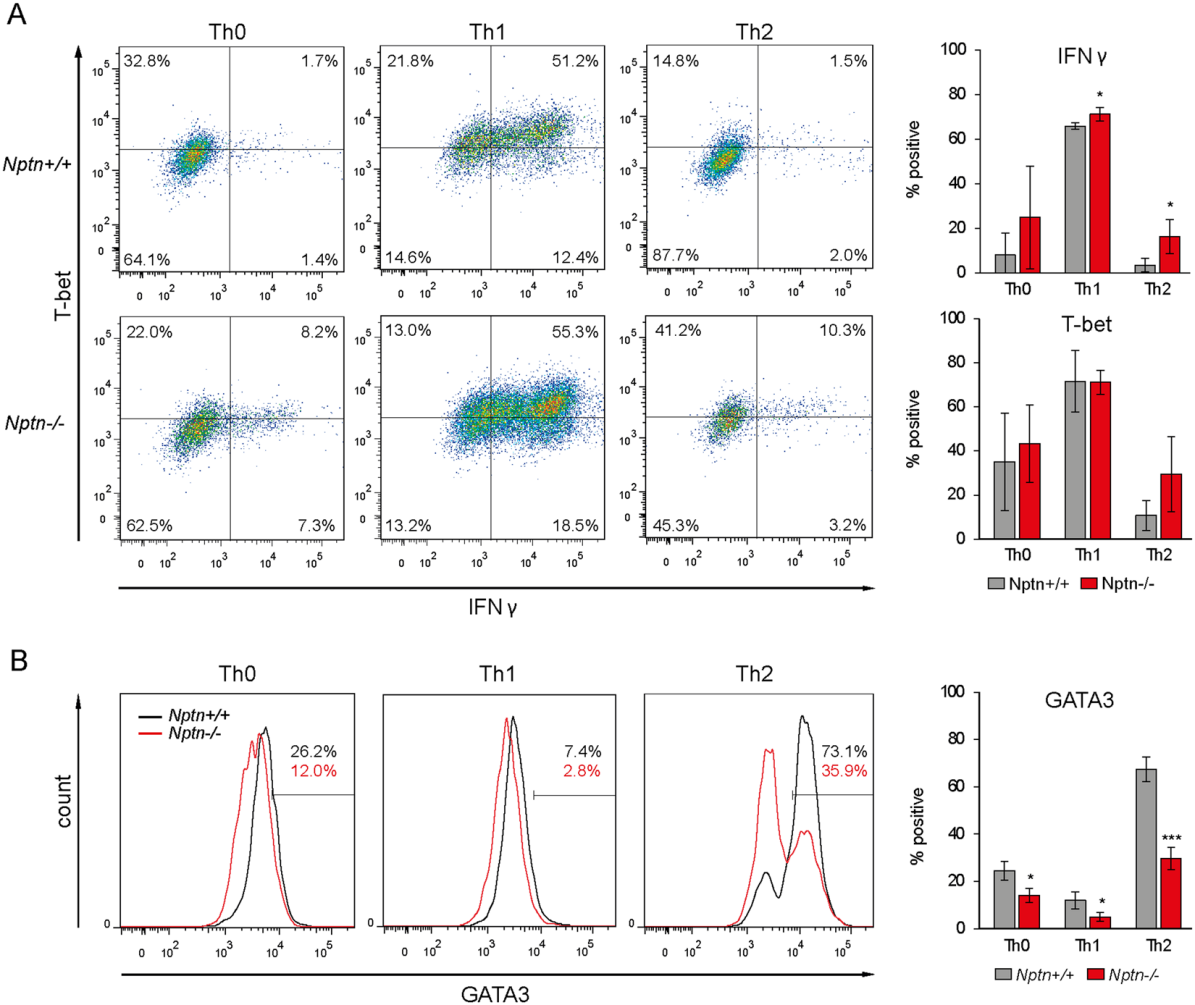
Polarization of *Nptn* ^−/−^ CD4 T cells favors Th1 differentiation. Expression of IFNγ and transcription factors in differentiated T helper cells. Naive CD4 T cells were cultured on immobilized anti-CD3 and anti-CD28 in medium supporting either Th0, Th1, or Th2 differentiation. On day 5 of culture, expression of IFNγ, T-bet or GATA3 was assessed by intracellular FACS staining. (**A**) Representative plots showing IFNγ and T-bet fluorescence under particular differentiation conditions. Mean proportions of IFNγ or T-bet expressing cells ± SD from 4 *wt* and 3 *Nptn* ^−/−^ mice are shown. (**B**) Representative fluorescence histograms of GATA3 expression. Bar graphs show mean proportions of GATA3 expressing T cells ± SD from 4 *wt* and 3 *Nptn* ^−/−^ mice for each condition. *p < 0.05, ***p < 0.001, unpaired two-tailed t-test.

T cell polarization to Th1 or Th2 subtypes is governed by specific transcription factors and cytokines and is accompanied by subtype-specific Ca^2+^ profiles^7–10^. Therefore, we wanted to test if Th1/Th2 differentiation is affected by loss of Neuroplastin. The T-bet transcription factor is a critical regulator of Th1 cells and IFNγ expression, while high levels of GATA3 and repression of T-bet and IFNγ are required for Th2 cells and the induction of IL4^7^. *Wt* and *Nptn* ^−/−^ naïve CD4 T cells were isolated from lymph nodes and polarized *in vitro* to Th1 and Th2 cells using TCR/CD28 co-stimulation. Using flow cytometry, we analyzed *wt* T cells for intracellular IFNγ, T-bet and GATA3, and confirmed effective polarization to the expected Th subtypes (Fig. 6A and B). However, in *Nptn* ^−/−^ T cells, the number of IFNγ-producing cells was moderately but significantly increased, despite the already high levels of IFNγ under Th1 conditions (Fig. 6A). *Nptn* ^−/−^ T cells also showed strongly reduced levels of GATA3 under Th2 conditions, in conjunction with elevated levels of T-bet and IFNγ (Fig. 6B). Furthermore, they also produced less GATA3 under Th0 and Th1 conditions (Fig. 6B). Since Th2 cells from C57BL/6 mice produce only very low levels of IL4 we could not detect significant amounts of intracellular IL4 under the described conditions. Therefore, we measured the presence of IL4 in culture supernatants. No IL4 could be detected in all Th0 or Th1 cultures. Under Th2 conditions low amounts of IL4 were present in cultures from *wt* T cells but even less in cultures of *Nptn* ^−/−^ T cells (Supplementary Fig. S8). Thus, polarization of *Nptn* ^−/−^ T cells favors Th1. Collectively, these data indicate that a Neuroplastin-PMCA signaling module plays an essential role in adjusting physiological Ca^2+^ levels in CD4 T cells, leading to proper expression of transcription factors during Th1 and Th2 commitment and differentiation.

## Discussion

Neuroplastin has multiple functions in the nervous system^18, 22–25, 30^. Although the Np55 splice variant is widely expressed^21^, very little is known about *in vivo* roles for it outside the nervous system. Here, we provide the first report that (i) Neuroplastin physically associates with PMCAs and (ii) that this interaction underlies a previously unnoticed requirement for Neuroplastin in murine T cell activation. We showed that its loss in T cells resulted in a profound reduction of both PMCA isoforms along with reduced Ca^2+^ clearance following stimulation. Importantly, all aspects of impaired Ca^2+^ regulation were phenocopied by genetic reduction of PMCA1 levels. Of note, we did not detect SERCAs or mitochondrial Ca^2+^ uniporters in the here presented screen nor in a series of MS-based analyses of Neuroplastin-specific immunoprecipitates isolated from mouse brain samples under less stringent conditions. To our knowledge, particular roles for PMCA1 and 4 in murine T cells have not been addressed and studies on human T cells have focused on PMCA4. Our study uncovered a previously unreported localization of murine PMCA1 to the immune synapse. The fact that heterozygosity of *Pmca1* already resulted in altered Ca^2+^ homeostasis argues for a pivotal role of this isoform in murine T cells. Along this line, the elevated Ca^2+^ in both *Nptn* ^−/−^ and *Pmca1* ^+*/*−^ T cells was accompanied by increased activation of NFAT and NFAT-dependent cytokine production. Coexpression of PMCAs and Np55 in many cell types suggests that this complex is a universal module in Ca^2+^ signaling. In fact, we have also noticed reduction of PMCA in *Nptn* ^−/−^ brains, though to a lesser extent than in T cells^23^. Thus, Neuroplastin regulation of Ca^2+^ clearance *via* PMCA may explain some of its neuronal roles in synaptogenesis, long-term potentiation, and memory processes^22–25^.

How might Neuroplastin stabilize PMCAs? Contrasting with the ~75% reduction of PMCAs at the protein level, we did not detect altered PMCA transcript levels in *Nptn* ^−/−^ samples. Susceptability of PMCAs to proteolytic cleavage by calpain or by caspases 1 and 3 is well-established^11, 13^. However, these proteases affect PMCA isoforms differently and typically produce detectable subfragments, which we never observed. It is rather conceivable that Neuroplastin stabilizes PMCAs co- and/or post-translationally in a chaperone-like manner, i.e. by supporting proper folding and membrane insertion of PMCAs as a prerequisite for further trafficking and surface expression. Loss of Neuroplastin may in turn lead to increased removal of PMCA molecules by co-translational, ER-associated protein degradation (ERAD)^31^, a mechanism that may even operate on nascent polypeptide chains^32^. Indeed, this would explain why neither our immunofluorescence labelings nor cellular fractionations pointed to an accumulation of PMCAs within secretory pathway compartments in *Nptn* ^−/−^ cells. Interestingly, valosin-containing protein (VCP, also known as Transitional endoplasmic reticulum ATPase, p97 or CDC48), a major player in ERAD and quality control of protein folding^33^, was among the few interaction partners for Neuroplastin that we identified by mass spectrometry when samples were prepared under stringent conditions (Supplementary Fig. S3A). However, the actual impact of this interaction with respect to Neuroplastin-dependent stabilization of PMCAs remains to be evaluated.

With respect to the observed Ca^2+^ phenotypes in *Nptn* ^−/−^ cells, stabilization of PMCAs early in the biosynthetic pathway appears as the most relevant function of Neuroplastin. This does not exclude additional roles for Neuroplastin, e.g. in controlling the trafficking or localization to synaptic subdomains^15–17^ or surface stability of PMCAs. In the absence of Neuroplastin, some PMCA1 still reaches the surface and the immune synapse. In human T cells, the Neuroplastin paralog CD147 was recently shown to interact with PMCA4, however, it was neither required for stabilization of PMCA4 nor for Ca^2+^ extrusion^34^. Therefore, it remains elusive whether CD147 is required to maintain certain levels of PMCA in the absence of Neuroplastin.

We found that in a competitive situation, *Nptn*-deficient hematopoietic stem or precursor cells do not efficiently seed the thymus. A particular requirement for PMCAs in these cell types is suggested by high transcript levels, especially for PMCA4 (www.immgen.org, data set 10357833). Therefore, concomitant reduction in both PMCA isoforms due to loss of Neuroplastin may account for impaired development of common lymphoid precursor subpopulations. Despite the strong reduction of PMCA in *Nptn-*deficient T cells, the elevated cytosolic Ca^2+^ levels did not apparently interfere with thymic T cell development. We cannot rule out, however, that a lower signaling threshold during thymocyte selection may have led to alterations in the TCR repertoire, thereby eliminating T cells prone to even more severe Ca^2+^ phenotypes. Moreover, our data indicate that T cells cope with PMCA reduction, at least in part, by increased uptake of Ca^2+^ into intracellular stores.

While the number and activity of PMCAs determine the kinetics of Ca^2+^ clearance, one would expect that normal baseline levels would always be reached in truly resting cells, even when PMCA levels are strongly reduced as in the case of *Nptn* deficiency. However, *Nptn* ^−/−^ (and to a lesser extent *Pmca1* ^+*/*−^) T cells displayed a robust increase in baseline [Ca^2+^]_i._ This may reflect ongoing Ca^2+^ flux during T cell development and naïve T cell homeostasis, with the consequence to persistently challenge the limited capacity of the remaining PMCA molecules. Indeed, while routinely scanning the surface of antigen presenting cells, naïve T cells experience tonic TCR signaling, which has been implicated in the maintenance of T cell responsiveness to antigen^35^. With PMCAs reduced, activity of SERCA gains impact on Ca^2+^ clearance, consistent with the observed Ca^2+^ overload of both the ER and mitochondria^36^. Notably, preliminary assessment of SERCA protein levels did not reveal up- or down-regulation in the absence of Neuroplastin.

The mean baseline [Ca^2+^]_i_ was elevated by ~ 65% in *Nptn* ^−/−^ T cells, whereas, consistent with a milder PMCA reduction, it was elevated by only ~30% in *Pmca1* ^+/−^ T cells. Still, nuclear NFAT levels and TCR-induced IFNγ production were similarly increased in cells of either genotype. Also, for both genotypes we observed that the portion of naïve T cells with instantaneous [Ca^2+^]_i_ values far above mean was much larger than for *wt*. This observation would be consistent with more frequent fluctuations, possibly oscillations, within individual cells. In fact, it is well-established that frequent Ca^2+^ oscillations are particularly efficient in maintaining nuclear NFAT^4, 5, 37^. Moreover, recent studies on non-immune cells demonstrated that PMCAs actively shape SOCE-induced Ca^2+^ oscillations in an isoform-specific manner^38^ and silencing of PMCA1 and 4 was found to increase Ca^2+^ oscillations and nuclear translocation of NFATc1 in osteoclasts^39^. Thus, while our FACS-based Ca^2+^ measurements did not resolve oscillations, we assume that reduced PMCA levels in both *Nptn* ^−/−^ and *Pmca1* ^+/−^ T cells exert their effects on NFAT and cytokine production at least in part *via* increased Ca^2+^ oscillation.

Sustained post-stimulation Ca^2+^ signals have been linked to T cell pathologies such as systemic lupus erythematosus^40, 41^, as well as to physiological determination of T helper cell fate towards Th1. In fact, TCR signaling strength, engendered through stable synapse formation and the presence of activating co-receptors on the APC, determines Ca^2+^ levels and T cell polarization outcomes^9, 42^. The role of Ca^2+^ in activating NFAT is canonical^6^, and constitutive NFAT signaling induces Th1 cell polarization^43^. Moreover, NFATc2 activation leads to IFNγ transcription and to T-bet activation^44–46^, leading to a Th1 differentiation program. The findings we report here affirm that increased Ca^2+^ levels play a major role in directing immune responses by polarizing activated T cells. In summary, our results define Neuroplastin as an essential partner for PMCAs and thus as a potential target for drug therapies on immune diseases related to impaired Ca^2+^ homeostasis in T cells.

## Methods

### Mice

*Nptn* ^−/−^ mice were backcrossed for more than 10 generations onto a C57BL/6 background^23^. Mice with a T cell receptor specific for ovalbumin peptide were generated by intercrossing with OT-II transgenic mice^47^. *Atp2b1* ^*tm1a(KOMP)Wtsi*^ mice were obtained from the UC Davis Knockout Mouse Project (www.komp.org; project ID CSD77635). *Atp2b1* ^*tm1a(KOMP)Wtsi*^ mice were crossed with a FLPo deleter mouse (*B6.Cg-Tg(Pgk1-flpo)10Sykr/J*, The Jackson Laboratory) to obtain *Atp2b1* ^*tm1c(KOMP)Wtsi*^ mice, referred here to as *Pmca1* ^*f/*+^ mice. T cell-specific excision was achieved by intercrossing *Pmca1* ^*f/*+^ with pTα^iCre^ mice^48^. Mice were housed in specific-pathogen-free conditions according to institutional guidelines. All procedures were performed in accordance with the institutional guidelines for health and care of experimental animals and were approved by the Landesverwaltungsamt Halle (representing the state of Saxony-Anhalt), Germany (Licence: 2-1181).

### Antibodies

A complete list of all antibodies used is provided in the Supplementary Methods.

### Immune cell preparation

For analysis of primary immune cells, mice were sacrificed in CO_2_ atmosphere, and thymi, spleens, lymph nodes, and femurs were dissected. Erythrocytes in spleen and bone marrow samples were lysed in 0.16 mM ammonium chloride solution. Cell numbers were determined by flow cytometry (FACS). Splenic or lymph node CD4 T cells and splenic B cells were enriched using the MACS CD4 T Cell Isolation Kit or the MACS naïve T cell isolation Kit and MACS CD43 MicroBeads, respectively, for mouse tissues, and an AutoMACS Pro Separator (Miltenyi Biotec, Bergisch Gladbach, Germany) according to the manufacturer’s guidelines. For proliferation assays, T cells were labeled with 5 µM 5-(6)-Carboxyfluorescein-diacetat-succinimidylester (CFSE, Sigma). For biochemical assays, cell pellets were frozen in liquid nitrogen and stored at −80 °C until further use.

### T cell culture

CD4 T cells were cultured in RPMI1640 with 10% FCS (Pan), 1xGlutamax, 1 mM NaPyruvate, non-essential amino acids, Penicillin/Streptomycin, 100 μM β-Mercaptoethanol (Gibco), referred to as R10 medium, on 96-well round bottom plates coated with 5 μg/ml anti-CD3 (2C11). For T cell – DC coculture, bone marrow derived dendritic cells (BMDC) were generated by culturing bone marrow cells in R10 medium, in the presence of 10% conditioned medium of AG8652 myeloma cells transfected with murine GM-CSF cDNA, as a source of GM-CSF. Medium was exchanged every third day. After 9 days of culture, maturation was induced by addition of 200 ng/ml LPS (Sigma) for further 24 h. Mature BMDC were incubated with 0.1 µM Ovalbumin peptide (pOva) (JPT Peptide Technologies, Berlin, Germany) 1 h before starting coculture experiments. 1 × 10^5^ OT-II transgenic CD4 T cells were cocultured with different numbers of mature, antigen-loaded BMDC in R10 medium in 96 well round bottom plates for 3 to 5 days. For intracellular cytokine detection, 50 ng/ml PMA, 1 μg/ml ionomycin, and 2 μg/ml Brefeldin A (Sigma) were added 4 h before analysis. For Th cell differentiation, naïve CD4 T cells were cultured on flat bottom 96-well plates precoated with 1 µg/ml of each anti-CD3 and anti-CD28 in IMDM medium (Gibco) supplemented as for R10 w/o Glutamax (cIMDM) alone for Th0 or with 20 ng/ml IL12 (PeproTech) and 10 ng/ml anti-IL4 for Th1, or with 10 ng/ml IL4 (R&D Systems) and 10 µg/ml anti-IFNγ for Th2 condition. After 3 days of culture, cells were restimulated for 2 days on freshly coated wells in cIMDM plus 5 ng/ml mouse IL2 (Biolegend). For the last 4 h of culture 10 ng/ml PMA, 1 μg/ml ionomycin and 10 μg/ml Brefeldin A were added. Intracellular cytokine staining was performed using the BD Fixation/Permeabilization Solution Kit or, in case of simultaneous detection of transcription factors, the Fixation/Permeabilization Kit (ebioscience). Stained cells were acquired on a BD FACSCanto II flow cytometer, and analyzed using FlowJo (Treestar). IL4 concentrations in culture supernatants were measured using the LEGENDplex Multi-Analyte Flow Assay Kit (Biolegend) according to the manufacturer’s guidelines.

### Ca^2+^ measurements

T cells from different donors were labeled separately with anti-CD4 using donor-specific fluorescent conjugates and mixed for further treatments and Ca^2+^ measurements within the same tube. Anti-CD62L was used to gate naïve T cells. For TCR-specific stimulation, T cells were prelabeled with 10 μg/ml hamster anti-CD3 (2C11). Cells were loaded with 1.3 μg/ml Fluo-3 and 2.7 μg/ml FuraRed (Life Technologies) in RPMI for 30 min at 37 °C, and incubated in standard Ringer’s solution (155 mM NaCl, 4.5 mM KCl, 2 mM MgCl2, 10 mM D-glucose, 5 mM Hepes, pH 7.4) which either contained 1 mM CaCl_2_ or 1 mM EGTA instead for measurements of Ca^2+^ release from internal stores. Fluo-3/FuraRed ratio was acquired on a BD FACSCanto II. TCR-specific response was induced by CD3 crosslinking with 10 μg/ml of anti-hamster F(ab’)_2_. Unspecific Ca^2+^ release was induced by 2 μg/ml ionomycin. Ca^2+^ release from the ER was induced by 1 μM SERCA blocker thapsigargin (Millipore). Ca^2+^ clearance following SOCE was investigated after treatment with thapsigargin as above and subsequent EGTA removal by washing and resuspension in Ca^2+^-containing Ringer’s solution. High Ca^2+^ levels resulting from SOCE were recorded for 3 min. To monitor Ca^2+^ clearance, cells were centrifuged and resuspended in Ca^2+^-free Ringer’s solution and acquired for 5 min. For direct assessment of SOCE after thapsigargin treatment, cells were first washed with EGTA-containing buffer, then washed and resuspended in Ca^2+^- and EGTA-free buffer. After recording Ca^2+^ baseline and release from the ER by thapsigargin, Ca^2+^ influx was induced by addition of an equal volume of Ringer solution containing 2 mM Ca^2+^, resulting in 1 mM final [Ca^2+^]_ex_, and acquired for 10 min. Ca^2+^ kinetics were analyzed using FlowJo and Excel. Instantaneous single cell ratios from different experiments were summarized as mean ± SD. Mean baseline ratios were averaged over 30 s and normalized to the average *wt* ratio for each experiment. Ca^2+^ decay phases were analyzed by fitting an exponential function to the mean ratios using Prism software. Since exponential decay after TCR-induced Ca^2+^ peak levels is delayed due to simultaneous influx and efflux, the best fit one-phase exponential regression was performed starting 1 min after the mean maximum ratio. For the rapid Ca^2+^ decay after thapsigargin-induced SOCE, the best fit one-phase exponential regression was performed for the first 30 s after removal of Ca^2+^. The fitted curve was calculated as Y = P + (Y_0_ − P) * EXP(−K * t) with Y_0_: ratio at t = 0, P: plateau ratio, K: rate constant [1/s], half time: ln(2)/K [s]. The initial rate was calculated at t = 0 from the first derivation of the fitted function with initial rate = −K * (Y_0_ − P).

### Immune synapse formation and immunofluorescence

For immune synapse formation, OT-II transgenic *wt* or *Nptn* ^−/−^ CD4 T cells were stimulated on immobilized anti-CD3 (5 μgl/ml) and anti-CD28 (1 μg/ml) for 3 to 5 days. B cells were stimulated overnight with 10 μg/ml LPS (Sigma) and loaded with 50 μM pOva (JPT). T and B cells were mixed 1:1, centrifuged 90 s at 300xg, carefully resuspended in R10 and incubated for 90 min at 37 °C on poly-L-lysine coated cover slips for synapse formation. T-B pairs were fixed with 4% PFA/ 5% sucrose and labeled overnight with anti-Neuroplastin in BD Perm/Wash buffer and subsequently with anti-PMCA1 in 0.05% Triton/ 10% donkey normal serum for 2 h followed by staining with donkey secondary antibodies and DAPI. Immune synapses were imaged on a Leica TCS SP5 confocal microscope.

For immunofluorescent NFATc2 and DAPI staining of CD4 T cells, we used the Fixation/Permeabilization Kit (ebioscience). For optimized comparability, *wt* and *Nptn* ^−/−^ cells were first separately labeled with anti-CD4 and mixed as described above. Confocal images with maximal DAPI signal were captured to determine ROIs for nuclear NFAT quantification using ImageJ. Nuclear NFAT intensities were normalized to the mean of *wt* cells for each experiment.

### Preparation of protein extracts from thymocytes, CD4 T cells and BMDM

For Western blots cell pellets were homogenized by incubation at 4 °C for 30 min in Triton-homogenization buffer (20 mM Tris, 50 mM NaCl, 1% Triton-X-100, pH 7.5, 2 mM MgCl_2_, 750 U/ml Benzonase (Sigma), containing protease inhibitors). After centrifugation at 15000xg for 20 min, supernatants were used for analysis.

### Preparation of Triton X-100 solubilized membrane proteins

Spleens and thymi from adult mice (postnatal age 10–20 weeks) were homogenized in saccharose HEPES buffer (320 mM Saccharose, 5 mM HEPES, pH 7.4, containing protease inhibitors (Roche). The homogenate was centrifuged at 1000xg for 10 min to remove nuclei and debris. Supernatant was centrifuged for 1 h at 100000xg. Pellets were washed in homogenization buffer (25 mM Tris, 500 mM NaCl, pH 7.4 including protease inhibitors) and centrifuged as above. The resulting pellet was then rehomogenized in the same buffer containing 0.5% Triton X-100. After incubation at 4 °C for 1 h the material was centrifuged at 20800xg for 1 h.

Total brains from adult mice were homogenized in homogenization buffer, centrifuged at 100000xg for 1 h and washed once. The pellet was then rehomogenized in buffer containing 0.5% Triton X-100. After 1 h incubation samples were spun again as above.

### Deglycosylation assay

Triton X-100 solubilized membrane proteins from spleen, thymus and total brain were ethanol precipitated, and deglycosylation was then performed using the Glyco Profile IV chemical deglycosylation kit (Sigma). Deglycosylated protein was purified by dialysis (Slide-A-Lyzer dialysis casette, 3.500 MWCO, ThermoScientific).

### Immunoprecipitation

Cell pellets were homogenized by carefull pipetting in Digitonin homogenization buffer (20 mM Tris, 50 mM NaCl, 1% Digitonin, pH 7.5, 2 mM MgCl_2_ and Benzonase (Sigma), containing protease inhibitors) incubated at 4 °C for 30 min and spun at 15000xg for 20 min. The resulting supernatant was precleared by 30 min incubation with ProteinG Sepharose^TM^ 4 Fast Flow (GE Healthcare). The lysate was then incubated with Neuroplastin antibody overnight. Protein G Sepharose beads were added for 2 h at 4 °C. Beads were washed three times with washing buffer (20 mM Tris, 150 mM NaCl, 0.5% Digitonin, pH 7.5) followed by a short rinse in 20 mM Tris/150 mM NaCl. For SDS-PAGE, bound proteins were eluted with 1x Rotiload (Roth). For mass spectrometry, beads were washed three times with PBS and finally resuspended in 50 mM ammonium bicarbonate.

### SDS-PAGE, Western blotting and quantitative Western Blot analysis

Protein content in cell lysates was determined using a bicinchoninic acid (BCA) kit according to the manufacturer’s instructions (Thermo Fisher Pierce). Samples were solubilized in sample loading buffer (RotiLoad, Roth, Germany) at concentrations of approximately 1.5 μg/μl and run on 4–20% SDS-Polyacrylamide gels. For immunodetection proteins were transfered onto nitrocellulose and incubated with the primary antibodies as indicated. Immunoreactivity was detected according to standard protocols using an ECL Imager (GeneGnome XRQ, Syngene, Cambridge, UK) or ECL-films. Images below saturation or films with the shortest exposure time still showing all expected bands were used for quantification by Image-J. For each blot, band intensities were normalized relative to the respective loading control and statistically analyzed using Prism software.

### Mass spectrometry

Neuroplastin immunoprecipitate beads were washed with PBS and resuspended in 50 mM ammonium bicarbonate. Cysteins were reduced with 2 mM dithiothreitol for 30 min at room temperature and subsequently ß-methylthiolated by addition of 10 mM methyl methanethiosulfonate. Digestion was performed by addition of 0.5 µg trypsin (Promega) and incubation overnight at 37 °C. Peptides were extracted by pooling the primary supernatant and the supernatant of a subsequent washing step using 0.1% (v/v) trifluoroacetic acid (TFA).

Peptides were purified with reversed-phase C18 ZipTip nano-columns (Millipore), eluted with 0.1% TFA/ 70% ACN, and dried. Protein identification was performed by high-resolution mass spectrometry on a hybrid dual-pressure linear ion trap/orbitrap mass spectrometer (LTQ Orbitrap Velos Pro, Thermo Scientific) equipped with an EASY-nLC Ultra HPLC (Thermo Scientific). For analysis, peptide samples were adjusted to 10 µl 0.1% TFA/ 1% ACN and fractionated on a 75 µm (ID), 25 cm PepMap C18-column, packed with 2 µm resin (Dionex/Thermo Scientific). Separation was achieved through applying a gradient from 2 to 35% ACN in 0.1% formic acid over 150 min at a flow rate of 300 nL/ min. An Orbitrap full MS scan was followed by up to 15 LTQ MS/MS runs using collision-induced dissociation (CID) fragmentation of the most abundantly detected peptide ions. Essential MS settings were as follows: full MS (FTMS; resolution 60 000; m/z range 400–2000); MS/MS (Linear Trap; minimum signal threshold 500; isolation width 2 Da; dynamic exclusion time setting 30 s; singly charged ions were excluded from selection). Normalized collision energy was set to 35%, and activation time to 10 ms. Raw data processing and protein identification were performed using PEAKS Studio V.8.0 (Bioinformatics Solutions). False discovery rate was set to <1%.

### Data Availability

All datasets generated and analyzed during the current study are available from the corresponding authors on reasonable request.

## Electronic supplementary material

Supplementary Information
