# Supplementary material for: A complex of Neuroplastin and Plasma Membrane Ca2+ ATPase controls T cell activation
Source: Sci Rep. 2017 Aug 21;7:8358. doi: 10.1038/s41598-017-08519-4 (PMC5566957; doi:10.1038/s41598-017-08519-4)
Supplement: Supplementary file 1 — Supplementary Information [file 41598_2017_8519_MOESM1_ESM.pdf]

Supplementary Information

A complex of Neuroplastin and Plasma Membrane Ca^{2+} ATPase controls T cell activation

Mark Korthals[1,#], Kristina Langnaese[1,#], Karl-Heinz Smalla[2,5,#], Thilo Kähne[3], Rodrigo Herrera-Molina[2], Juliane Handschuh[2], Anne-Christin Lehmann[1], Dejan Mamula[1], Michael Naumann[3], Constanze Seidenbecher[2,5], Werner Zuschratter[4], Kerry Tedford[1], Eckart D. Gundelfinger[2, 5-7], Dirk Montag[8], Klaus-Dieter Fischer[1,*] , Ulrich Thomas[2,*]

[1] Institute of Biochemistry and Cell Biology, Otto-von-Guericke-University, Medical Faculty, D-39120 Magdeburg, Germany

[2] Department of Neurochemistry and Molecular Biology, Leibniz Institute for Neurobiology, D-39118 Magdeburg, Germany

[3] Institute of Experimental Internal Medicine, Otto-von-Guericke-University, Medical Faculty, D-39120 Magdeburg, Germany

[4] Special Lab Electron and Laserscanning Microscopy, Leibniz Institute for Neurobiology, D-39118 Magdeburg, Germany

[5] Center for Behavioral Brain Sciences, D-39120 Magdeburg, Germany

[6] Medical Faculty, Otto von Guericke University, D-39120 Magdeburg, Germany

[7] German Center for Neurodegenerative Diseases (DZNE) Site Magdeburg, D-39120 Magdeburg, Germany.

[8] Neurogenetics Special Laboratory, Leibniz Institute for Neurobiology, D-39118 Magdeburg, Germany

[#] M.K., K.L., K.H.S. These authors contributed equally to this work.

[*] K.D.F., U.T. equally contributing corresponding authors.

Correspondence:

Ulrich.Thomas@lin-magdeburg.de ++49 (0)391 6263 93231

klaus.fischer@med.ovgu.de ++49 (0)391 6714256

Supplementary Figure Legends

Supplementary Figure S1. Normal T cell development and unaltered TCR-induced proliferation, early activation marker expression and MAPK activation in the absence of Neuroplastin.

(**A**) FACS analysis of T cell populations in mixed bone marrow chimeras. Ly5.2^{+} *wt* or *Nptn*$^{-/-}$ and competitive Ly5.1^{+} donor cells were distinguished with anti-Ly5.2 and anti-Ly5.1 antibodies, respectively. Bar graphs represent the proportions of Ly5.2^{+} *wt* or *Nptn*$^{-/-}$ cells in thymus, lymph nodes and spleen, and respective frequencies of T cell populations within Ly5.2^{+} *wt* or *Nptn*$^{-/-}$ cells, respectively. Mean values ± SD with n ≥ 3, *p < 0.05, ***p < 0.001. (**B**) Proliferation of *wt* and *Nptn*$^{-/-}$ T cells assessed by CFSE dilution. CFSE-labeled CD4 T cells were cultured for 3 days on different doses of immobilized anti-CD3 and analyzed by FACS. Representative histograms showing CFSE dilution from one of two independent experiments are presented. Small numbers indicate completed cell cycles as determined by half reduction of CFSE intensity. (**C**) FACS analysis of CD69 surface expression after stimulation for different time intervals on immobilized anti-CD3. Mean proportions of CD69^{+} cells ± SD were summarized from two independent experiments. (**D**) Intracellular FACS of MAPK activation. T cells were labeled with anti-CD3 (2C11) and stimulated with a CD3 crosslinking antibody for different time intervals before intracellular phospho-Erk1/2 (Thr202/Tyr204) staining. The diagram shows kinetics of the mean proportions of phospho-Erk^{+} cells ± SD from 4 *wt* and 2 *Nptn*$^{-/-}$ mice.

Supplementary Figure S2. *Nptn*$^{-/-}$ phenotypes are T cell intrinsic

(**A**) Ca^{2+} baseline levels in *wt* and *Nptn*$^{-/-}$ T cells from mixed bone marrow chimeras. Anti-Ly5.1 and anti-Ly5.2 antibodies were used to distinguish T cells from different donors. For ratiometric Ca^{2+} measurement by FACS, cells were gated on CD62L^{+} naïve T cells, and Fluo-3/ FuraRed ratio was recorded for 4 min in Ca^{2+}-containing buffer. Histograms represent the distribution of instantaneous baseline ratios in single Ly5.2^{+} *wt* or *Nptn*$^{-/-}$ naive T cells (more than 30.000 cells each), respectively, compared to corresponding Ly5.1^{+} control cells derived from two chimeric mice. (**B**) Nuclear NFAT intensities in T cells from chimeric mice were measured by confocal imaging. Data were separately quantified for Ly5.2^{+} and Ly5.1^{+} cells, respectively, and normalized to the mean nuclear NFAT intensity of the corresponding Ly5.1^{+} cells from each individual chimeric mouse. Normalized values of all 444 Ly5.2^{+} *wt* with 246 corresponding Ly5.1^{+} control cells and 393 Ly5.2^{+} *Nptn*$^{-/-}$ with 342 corresponding Ly5.1^{+}

control cells from 3 experiments are shown. Overlay bars represent the mean normalized values ± combined SD from these 3 experiments, *** $p < 0.001$, unpaired two-tailed t-test.

Supplementary Figure S3. MS of Neuroplastin immunoprecipitates from thymocytes.
(**A**) Full MS list of significant Neuroplastin interaction partners in thymocytes. Protein composition of Neuroplastin-immunoprecipitates (IP) of *wt* mouse thymocytes (n=3) was resolved by mass spectrometry and controlled by IPs from *Nptn*$^{-/-}$ thymocyte samples (n=4). The list exclusively shows confidently identified proteins (-10logP>20). All proteins identified in Neuroplastin-IPs from *Nptn*$^{-/-}$ samples were considered as unspecific and removed from the list. (**B**) Alignments of PMCA1 (UniProt entry name AT2B1), PMCA2 (AT2B2), PMCA3 (A2ALL9) and PMCA4 (AT2B4) proteins. Peptides identified by LC-MS/MS in Neuroplastin-IPs from thymocyte samples are highlighted in blue if common to more than one isoform and in yellow if unique to one particular isoform.

Supplementary Figure S4. Neuroplastin Np55 expression in Jurkat T cells and localization at the immune synapse.
Confocal images of immune synapses formed between Jurkat T cells expressing GFP-tagged rat Np55 and SEE-loaded Raji B cells. Pairs of T cells and B cells were fixed and immunofluorescently labeled with an anti-CD3 antibody. The DIC image shows the orientation of the T-B pair. The 3D reconstruction of confocal images shows accumulation of Np55-GFP signals (green) at the periphery and of CD3 (magenta) in the center of the immune synapse.

Supplementary Figure S5. Neuroplastin-PMCA interaction in bone marrow derived macrophages.
(**A**) Verification of the Neuroplastin-PMCA complex by Western blot detection of PMCA1 and 4 in Neuroplastin-IPs from lysates of bone marrow derived macrophages (BMDM). Asterisks mark immunoglobulin light and heavy chain bands. *Nptn*$^{-/-}$ controls confirmed specificity. (**B**) Western blot analysis of PMCA1, PMCA4 and Neuroplastin in *wt* and *Nptn*$^{-/-}$ BMDM. Representative blots show lack of Neuroplastin and strong reduction of PMCAs in *Nptn*$^{-/-}$ BMDMs. β-actin served as a loading control for normalization. Bar graphs show normalized intensities ± SEM from 4 *wt* and 4 *Nptn*$^{-/-}$ replicate samples analyzed on the same blot *: $p < 0.05$, **: $p < 0.01$, unpaired two-tailed t-test. (**C**) qRT-PCR analysis of PMCA1 reveals normal transcript levels in *Nptn*$^{-/-}$ BMDMs. *B2m* and *Hprt* transcripts served as

references for normalization. Graph shows mean ± SD for n=5 per group. (**D**) Verification of Neuroplastin-PMCA1 interaction on the surface of BMDM. Neuroplastin-IPs were prepared after surface biotinylation of intact *wt* and *Nptn*$^{-/-}$ BMDMs. Immunoprecipitated proteins were then affinity-purified on avidin columns to enrich for biotinylated surface proteins. Western blots show that PMCA1 co-immunoprecipitates with Neuroplastin on the cell surface of *wt* cells. Samples from *Nptn*$^{-/-}$ mice served as a negative control. (**E**) Remaining PMCA1 in *Nptn*$^{-/-}$ cells is expressed at the cell surface. Cell surface proteins were biotinylated as in (D) but affinity-purified from total protein lysates. Western blot analysis shows the presence of PMCA1 at the surface of both *wt* and *Nptn*$^{-/-}$ cells. Unbiotinylated cells served as a negative control. (**F**) Subcellular distribution of PMCA1 remains largely unaffected by loss of Neuroplastin. OptiPrep gradient fractionation (24 fractions) derived from *wt* and *Nptn*$^{-/-}$ BMDM extracts were analyzed by Western blots probed with antibodies against Neuroplastin, PMCA1 and a selection of established compartment markers. Analysis reveals a similar subcellular distribution of PMCA1 in *wt* and *Nptn*$^{-/-}$ BMDM (2nd versus 3rd panel). The distribution of Neuroplastin is shown for comparison (upper panel). Note co-distributions of the 45 kDa form of Neuroplastin with Grp78 and of highly glycosylated Neuroplastin with PMCA1.

Supplementary Figure S6. Normal store operated Ca^{2+} entry (SOCE) in *Nptn*$^{-/-}$ T cells
Flow cytometric ratiometric measurement of SOCE in *wt* or *Nptn*$^{-/-}$ naïve CD4 T cells. After baseline recording, SERCA was blocked by the addition of 1 µg/ml thapsigargin in Ca^{2+} - and EGTA-free buffer. After monitoring Ca^{2+} release from the ER and return to baseline levels, Ca^{2+} influx was induced by addition of 1 mM Ca^{2+}. Mean ratios ± SD of *wt* and *Nptn*$^{-/-}$ T cells from two experiments are shown.

Supplementary Figure S7. Haploinsufficiency of PMCA1 in CD4 T cell function.
(**A**) Western blot analysis of PMCA1 and PMCA4 in *wt* unstimulated CD4 T cells and lymphoblasts (30 µg/lane). Both proteins are upregulated by stimulation on immobilized anti-CD3 plus anti-CD28. (**B**) Western blot analysis reveals reduced PMCA1 levels in *Pmca1*$^{f/+}$ *cre*$^{+}$ thymocytes compared to *wt* thymocytes (30 µg/lane). β-actin served as loading control for normalization. Bar graphs show mean normalized PMCA1 signal intensities ± SEM from 4 *wt* (*cre*$^{-}$) plus 4 *Pmca1*$^{f/+}$ (*cre*$^{+}$) replicate samples analyzed on the same blot *: $p < 0.05$, unpaired two-tailed t-test. (**C**) Flow cytometric ratiometric measurement of TCR-induced Ca^{2+} flux in naive T cells from *Pmca1*$^{f/+}$ mice as performed in Fig. 2A. Mean Fluo-3/FuraRed

ratios ± SD in cells from 3 *cre*$^-$ and 2 *cre*$^+$ mice are shown. (**D**) The distribution of different instantaneous Ca^{2+} baseline levels in single *wt* and *Pmca1*$^{f/+}$*(cre*$^+$*)* T cells is shown as a histogram of the Fluo-3/FuraRed ratios acquired over 30s. (**E**) Baseline Ca^{2+} levels of T cells from *Pmca1*$^{f/+}$ mice were measured in the same way as shown in Fig. 2A. Bar graphs present mean normalized ratios ± combined SD derived from 5 experiments. *** $p < 0.001$. Further quantifications of baseline and peak levels as well as the decay phase are summarized in Supplementary Table 2. (**F**) Quantification of nuclear NFAT intensity in T cells from *Pmca1*$^{f/+}$ mice as performed in Fig. 2B. Normalized values of all 1149 *cre*$^-$ and 789 *cre*$^+$ T cells from 4 experiments are presented with overlay bars showing the mean normalized values ± combined SD, *** $p < 0.001$, unpaired two-tailed t-test. (**G**) Intracellular detection of IFNγ in stimulated CD4 T cells from *Pmca1*$^{f/+}$ mice as in Fig. 2C. IFNγ levels in viable cells stimulated for 6 days on immobilized anti-CD3 are shown as representative fluorescence histograms. The proportions of IFNγ producing cells at day 6 were summarized as mean ± SD from 4 *cre*$^-$ and 5 *cre*$^+$ mice, * $p < 0.05$, unpaired two-tailed t-test.

Supplementary Figure S8. Reduced IL4 secretion in *Nptn*$^{-/-}$ T cells

Secretion of IL4 in differentiated T helper cells. Naive CD4 T cells were cultured on immobilized anti-CD3 and anti-CD28 in medium supporting either Th0, Th1, or Th2 differentiation. IL4 was measured and quantified flow cytometrically in supernatants from day 5 cultures. The bar graph shows the mean IL4 concentration ± SD produced by *wt* and *Nptn*$^{-/-}$ T cells from 3 independent experiments. *: $p < 0.05$, paired two-tailed t-test.

Supplementary Methods

**Complete list of antibodies**						
**Primary antibodies**						
**Target**	**Clone**	**Host**	**Label***	**Application**	**Dilution**	**Company**
β-actin	AC-15	mouse	unlabeled	WB	1:10000	Sigma
BiP/GRP78	40/BiP	mouse	unlabeled	WB	1:1000	BD
CD3ε	145-2C11	Armenian hamster	unlabeled/ LEAF	stimulation	variable	Biolegend
human CD3	UCHT1	mouse	unlabeled	IF	1:1000	eBioscience
CD4	RM4-5	rat	Fluo	FC,<br>FC Ca	1:200<br>1 µg/ml	Biolegend, BD
CD8	53-6.7	rat	Fluo	FC	1:200	Biolegend
CD11b	M1/70	rat	Fluo	FC	1:100	Biolegend, BD
CD44	IM7	rat	Fluo	FC	1:200	Biolegend
CD45.1/Ly5.1	A20	mouse	Fluo	FC,<br>FC Ca,<br>IF	1:200<br>1µg/ml<br>1:100	Biolegend, BD
CD45.2/Ly5.2	104	mouse	Fluo	FC,<br>FC Ca,<br>IF	1:200<br>1 µg/ml<br>1:100	Biolegend, BD
CD45R/B220	RA3-6B2	rat	Fluo	FC	1:200	Biolegend
CD62L	MEL-14	rat	Fluo	FC,<br>FC Ca	1:200<br>1 µg/ml	Biolegend
CD69	H1.2F3	Armenian hamster	Fluo	FC	1:200	BD
EEA1	14/EEA1	mouse	unlabeled	WB	1:2500	BD
GATA3	L50-823	mouse	Fluo	ICFC	1:50	BD
GM130	35/GM130	mouse	unlabeled	WB	1:500	BD
IFNγ	XMG1.2	rat	Fluo<br>unlabeled/ LEAF	ICFC<br>blocking	1:100<br>10 µg/ml	BD<br>Biolegend
IL2	JES6-5H4	rat	Fluo	ICFC	1:100	Biolegend
IL4	11B11	rat	unlabeled/ LEAF	blocking	10 ng/ml	Biolegend
LAMP-1	polyclonal	rabbit	unlabeled	WB	1:700	Abcam
Na^+/K^+-ATPase	polyclonal	rabbit	unlabeled	WB	1:1000	Cell Signaling
Neuroplastin	polyclonal	sheep	unlabeled	FC<br>IF<br>WB<br>IP	1:40<br>1:100<br>1:4000<br>1 µg	R&D Systems
NFAT1 (NFATc2)	D43B1	rabbit	unlabeled	IF	1:100	Cell Signaling
phospho-Erk1/2	197G2	rabbit	unlabeled	ICFC	1:200	Cell Signaling
PMCA1	EPR12029	rabbit	unlabeled	IF<br>WB	1:100<br>1:5000	Abcam
PMCA4	JA9	mouse	unlabeled	WB	1:1000	Abcam
T-bet	O4-46	mouse	Fluo	ICFC	1:50	BD
TCR β-chain	H57-597	Armenian hamster	Fluo	FC	1:200	BD
**Secondary antibodies**						
**Target**	**Clone**	**Host**	**Label**	**Application**	**Dilution**	**Company**
Rabbit IgG	polyclonal	donkey	Alexa Fluor 647, Alexa Fluor 488	IF	1:500	Thermo Fisher

Sheep IgG	polyclonal	donkey	DyLight 488	FC, IF	1:200	AbD Serotec
Hamster IgG	polyclonal $F(ab')_2$	goat	unlabeled	stimulation	variable	AbD Serotec
Mouse IgG	polyclonal	goat	POD	WB	1:10000	Jackson ImmunoResearch
Rabbit IgG	polyclonal	goat	POD	WB	1:10000	Jackson ImmunoResearch
Mouse IgG	polyclonal	goat	Cy5	IF	1:500	Jackson ImmunoResearch
Sheep IgG	polyclonal	donkey	POD	WB	1:10000	Jackson ImmunoResearch

FC, flow cytometry; FC Ca, flow cytometric ratiometric Ca^{2+} measurement; ICFC intracellular flow cytometry; IF, immunofluorescence; IP, immunoprecipitation; LEAF, low endotoxin, azid-free; POD, horseradish peroxidase; WB, Western blot.
* Fluo, primary antibodies directly conjugated to one of the following fluorochromes: FITC, Alexa488, PE, PerCP, APC, V450, BV421, BV510, PE-Cy7, APC-Cy.

Bone marrow chimeric mice

For bone marrow transfer experiments, bone marrow cells obtained from either $Ly5.2^+$ ($CD45.2^+$) wildtype or *Nptn*$^{-/-}$ donor mice were mixed 1:1 with bone marrow cells from $Ly5.1^+$ donor mice. Recipient $Ly5.1^+$ (=$CD45.1^+$) mice were lethally irradiated with 10 Gy and *i.v.* injected with 100µl PBS containing $2x10^6$ mixed bone marrow cells. The repopulation of immune organs in chimeric mice was analyzed approximately 8 weeks after donor cell transfer. In Ca^{2+} measurements and nuclear NFAT imaging, cells from different donors were distinguished by labelling with anti-CD45.1 and anti-CD45.2 antibodies with different fluorescent conjugates.

Detection of TCR mediated MAPK activation

For TCR induced activation of phospho-Erk1/2, CD4 T cells were first labeled with 10 µg/ml hamster anti-CD3 (2C11), washed and resuspended in RPMI1640. Before stimulation, T cells were serum-starved for ~30 min at 37 °C / 5% CO_2. Stimulation was induced by crosslinking TCR molecules with 50µg/ml of an anti-hamster $F(ab')_2$ at 37 °C. After different time periods ranging from 0 to 10 min, cells were immediately fixed by adding 3% paraformaldehyde (PFA) for 20 min at room temperature. After washing, cells were permeabilized with 100% ice cold methanol for at least 30 min on ice, washed and rehydrated with PBS/0.2% BSA. Cells were then incubated with a rabbit anti-phospho-Erk1/2 antibody followed by staining with a donkey anti-rabbit secondary antibody and analyzed by FACS.

Differentiation of macrophages

Bone marrow derived macrophages (BMDM) were established from bone marrow cells cultured for 7 days on “bacteriological” plastic plates in bone marrow differentiation medium (DMEM GlutaMax supplemented with 10% FCS, 1x MEM non-essential amino acids, 50 µM

β-mercaptoethanol, Penicillin/Streptomycin, and 10% L929-cell conditioned medium as a source of M-CSF). Medium was exchanged after 4 days. On day 7 BMDM were detached by trypsinization, washed with PBS.

Expression of Np55-GFP in Jurkat T cells and pair formation with Raji B cells.

A construct for expression of C-terminally EGFP-tagged rat Np55 was generated using standard cloning protocols. In brief, the open reading frame of rat Np55 was PCR-amplified from a cDNA (Langnaese *et al*. 1997) with primers containing restriction sites for BamH1 on either end and inserted into the pEGFP-N3 vector (Clontech).

Jurkat T cells (JE6.1) and Raji B cells (ATCC) were maintained at $1x10^5$ cells/ml to $1x10^6$ cells/ml in RPMI1640 with 1xGlutamine (Biochrom), 1x Penicillin/Streptomycin (Gibco) and 10% FCS (Pan). A day prior to electroporation, 2.5 x 10^5/ml Jurkat cells were cultured in medium as above but without antibiotics. For electroporation, cells were washed and resuspended in PBS with Ca^{2+} and Mg^{2+}. 10^7 cells were then transferred to the electroporation cuvette and mixed with 10 µg of the Np55 cDNA construct. Electroporation was performed at 250V/950 µF using a Gene Pulser electroporator (Bio-Rad). Immediately after electroporation, cells were cultured for recovery in medium without antibiotics. Transfection efficiency and viability of the cells was checked at 24, 48, 72, 96 hours by FACS. Immune synapse formation was performed at the peak of transfection efficiency (usually 48 hours after transfection).

On the day before synapse formation, Raji cells were incubated overnight in RPMI medium supplemented with 10% FCS and 1µg/ml staphylococcal enterotoxin E (SEE, Toxin Technology) at 37°C. Superantigen-loaded Raji B cells and transfected Jurkat T cells were mixed 1:1 at $2.5x10^6$/ml, and 40 µl of the mixture was transferred on poly-L-lysine coated coverslips and incubated for 15min at 37°C. B-T pairs were fixed with 2% PFA for 15 min, permeabilized for 10 min with 0.1% Triton-X100, washed with PBS and blocked for 30 min in PBS with 2 % horse serum. Cells were the incubated over night with a mouse anti-human CD3 antibody (UCHT1) followed by 1h incubation with Cy5 anti-mouse secondary antibody in PBS / 2% serum and mounted on slides with Mowiol 4-88 (Calbiochem). Immune synapses were imaged on a Leica TCS SP5 confocal microscope.

Quantitative RT-PCR

Total RNA was isolated from mouse cells using peq-GOLD TriFast reagent (PeqLab, Erlangen, Germany) and DNase treated using the TURBO DNA-free Kit (Ambion, Austin,

USA). First strand cDNA was prepared with the RevertAid First Strand cDNA synthesis kit (Fermentas, St. Leon-Roth, Germany) using random primers. For preparation of minus-RT negative control the RT enzyme was ommited. Real-time PCR was performed on a MX3005P device (Stratagene, La Jolla, CA, USA). The reactions contained 1x Brilliant SYBR Green QPCR Master Mix (Stratagene), 30 nM ROX reference dye, 200 nM of each primer and prediluted cDNA (corresponding to 20 ng RNA) in a 25 µl reaction. Expression of *B2m* and *Hprt* as reference genes was analyzed in parallel. Primer sequences were as follows 5'-GTGGCCAGATCTTGTGGTTT-3' and 5'-CATCAATAAGGGGGATGTGC-3' for *Pmca1*, 5'-CTGCTACGTAACACAGTTCCACCC-3' and 5'-CATGATGCTTGATCACATGTCTCG-3' for *B2m*, *Hprt* 5'-CTCATGGACTGATTATGGACAGGAC-3' and 5'-GCAGGTCAGCAAAGAACTTATAGCC-3'. After initial denaturation at 95°C for 10 min, amplification was performed with 40 cycles of denaturation at 95°C for 30 s, annealing at 57°C for 40 s and extension at 72 °C for 40 s. No signals were detected in no-template controls and in minus-RT controls. The experimental threshold (C_t) was calculated using the MxPro Mx3005P v3.00 software. All samples were run in duplicate and the mean value of each duplicate was used for all further calculations. The relative expression level of *Pmca1* in each sample was calculated as described in detail previously (Langnaese *et al.*, 2008) and statistically analyzed using GraphPad Prism (GraphPad Software, Inc., San Diego, CA, USA, version 5.04).

OptiPrep™ gradient fractionation

Postnuclear supernatants (PNS) from BMDM were prepared essentially following the manufacturer's application shield NS3 (Axis-Shield, Oslo, Norway). Briefly, 10-12 plates (10 cm diameter) with near confluent BMDM were washed at room temperature with PBS, then with homogenization buffer (250 mM Saccharose, 10 mM triethanolamine, 10 mM acetic acid pH 7.8). Cells were scraped off with homogenization buffer containing 1 mM EDTA and protease inhibitors at 4 °C, collected by centrifugation at 300xg for 10 min and resuspended in the same buffer (5 volumes per one volume cell pellet). Homogenization was performed using a tight fitting Dounce homogenizer. The homogenate was centrifuged at 750xg for 10 min and the resulting supernatant again at 950xg for 10 min to obtain PNS. Continous 5-25% OptiPrep™ (iodixanol. Axis-Shield) gradients that were formed overnight at 4 °C from a 5/10/15/20/25% step gradient in homogenization buffer (application sheet NS1). PNS (1.6-2.5 mg protein/ gradient), adjusted to 2% OptiPrep, were layered on top of gradients and

centrifuged in a Sorvall TH641 rotor at 48000xg for 18 h (application sheet S20). Fractions were taken from top to bottom.

Linearity of the gradient was confirmed by measuring the absorption at 244 nm in small aliquots of each fraction. Proteins were concentrated by TCA precipitation and dissolved in sample buffer (1x Rotiload). Equal volumes of all fractions were run in SDS-PAGE, blotted onto nitrocellulose and probed with indicated antibodies.

Surface biotinylation assay

BMDM ($2x10^7$ cells) were surface biotinylated with 0.25 mg/ml EZ-Link Sulfo-NHS-SS-Biotin in PBS according to the manufacturer’s protocol (Pierce cell surface protein isolation kit, Thermo Scientific, Rockford, IL). Cells were then incubated at 4 °C for 30 min in lysis buffer (20 mM Tris, 150 mM NaCl, 1% Digitonin, pH 7.5, 2 mM $MgCl_2$, 750 U/ml Benzonase and protease inhibitors) and centrifuged at 15000xg for 30 min. Half of the lysate was directly incubated with NeutrAvidin Agarose for 3 h at 4 °C to isolate surface biotinylated proteins. After washing, beads were eluted with SDS-PAGE sample buffer containing 50 mM DTT (Avidin-pulldown). To specifically analyze the Neuroplastin complex, the remaining half of the lysate was subjected to immunoprecipitation with Neuroplastin antibody (antibody incubation restricted to 1.5 h). Three IPs, each on 1 mg lysate, were performed in parallel. After washing, protein G beads were boiled twice for 3 min with 20 µl 10% SDS. The combined IP-eluates were diluted with buffer containing 20 mM Tris, 150 mM NaCl, 0.5% Digitonin, pH 7.5, and protease inhibitors in order to yield 0.5% SDS, and then incubated with NeutrAvidin Agarose to isolate the biotinylated subfraction in the immunoprecipitate.

Supplementary Reference

Langnaese, K., John, R., Schweizer, H., Ebmeyer, U., and Keilhoff, G. (2008). Selection of reference genes for quantitative real-time PCR in a rat asphyxial cardiac arrest model. BMC molecular biology *9*, 53.

Langnaese, K., Beesley, P.W., Gundelfinger, E. D. (1997). Synaptic membrane glycoproteins gp65 and gp55 arenew members of the immunoglobuline superfamily. J Biol Chem. 10, 272(2), 821-7

Supplementary Fig. S1

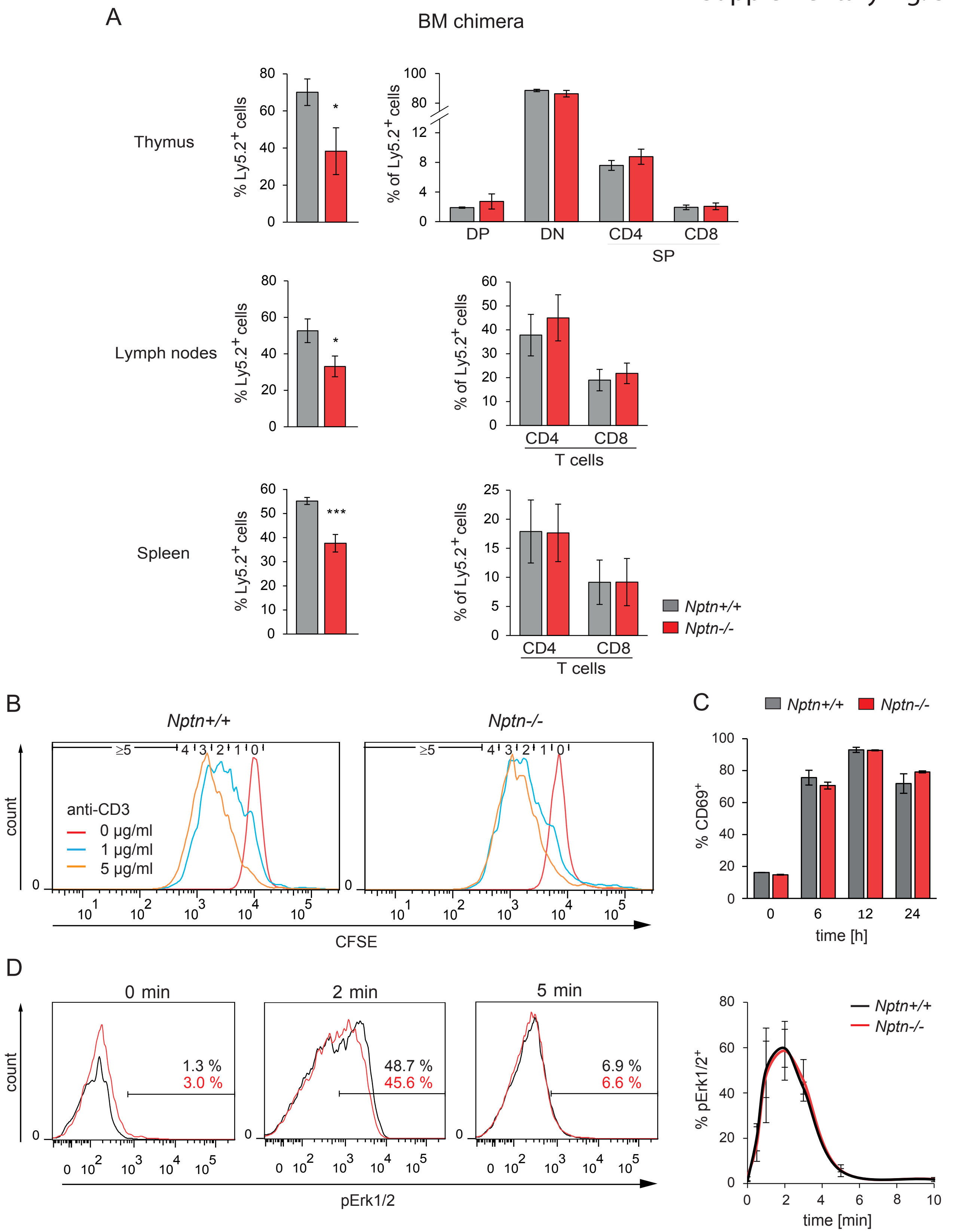

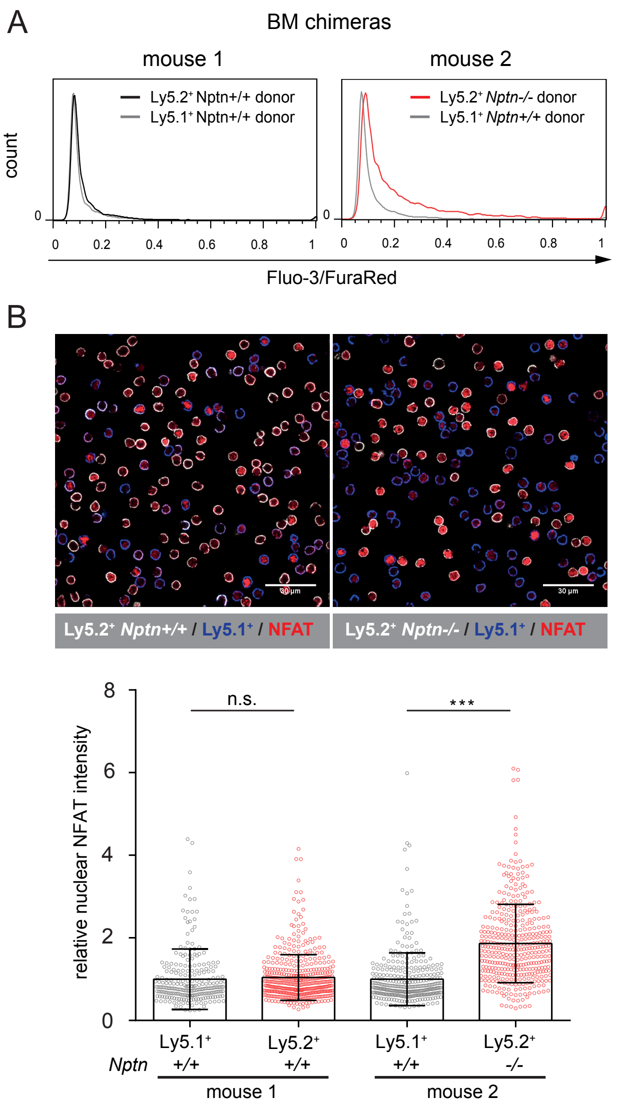
A
BM chimeras
mouse 1
mouse 2
Ly5.2+ Nptn+/+ donor
Ly5.1+ Nptn+/+ donor
Ly5.2+ Nptn-/- donor
Ly5.1+ Nptn+/+ donor
count
0
0.2
0.4
0.6
0.8
1
Fluo-3/FuraRed
B
30 µm
Ly5.2+ Nptn+/+ / Ly5.1+ / NFAT
Ly5.2+ Nptn-/- / Ly5.1+ / NFAT
relative nuclear NFAT intensity
8
6
4
2
0
n.s.
***
Ly5.1+
Ly5.2+
Nptn
+/+
-/-
mouse 1
mouse 2

A

Accession	Entry name	-10lgP	#Peptides	#Unique peptides	Coverage (%)	Description
G5E829	AT2B1_MOUSE	326.9	35	19	26.1	Plasma membrane calcium-transporting ATPase 1 OS=Mus musculus GN=Atp2b1 PE=1 SV=1
Q6Q477	AT2B4_MOUSE	269.9	18	7	14.1	Plasma membrane calcium-transporting ATPase 4 OS=Mus musculus GN=Atp2b4 PE=1 SV=1
Q01853	TERA_MOUSE	265.1	45	29	27.0	Transitional endoplasmic reticulum ATPase OS=Mus musculus GN=Vcp PE=1 SV=4
P97300	NPTN_MOUSE	173.7	8	8	18.2	Neuroplastin OS=Mus musculus GN=Nptn PE=1 SV=3
P53986	MOT1_MOUSE	162.3	7	6	20.6	Monocarboxylate transporter 1 OS=Mus musculus GN=Slc16a1 PE=1 SV=1
Q8C854	MYEF2_MOUSE	150.9	7	7	8.9	Myelin expression factor 2 OS=Mus musculus GN=Myef2 PE=1 SV=1
Q8JZQ2	AFG32_MOUSE	143.3	10	6	9.1	AFG3-like protein 2 OS=Mus musculus GN=Afg3l2 PE=1 SV=1
P57776	EF1D_MOUSE	125.6	5	5	13.5	Elongation factor 1-delta OS=Mus musculus GN=Eef1d PE=1 SV=3
Q60864	STIP1_MOUSE	119.0	7	7	8.0	Stress-induced-phosphoprotein 1 OS=Mus musculus GN=Stip1 PE=1 SV=1

B

```
AT2B1_MOUSE   MGDMANNSVAYSGVKNSLKEANHDGDFGITLTELRALMELRSTDALRKIQESYGDVYGIC
AT2B2_MOUSE   MGDMTNSDFY---SKNQRNESSHGGEFGCTMEELRSLMELRGTEAVVKIKETYGDTEAIC
A2ALL9_MOUSE  MGDMANSSIEFHPKPQQQREVPHVGGFGCTLAELRSLMELRGAEALQKIQEAYGDVSGLC
AT2B4_MOUSE   ---MTNPPG--QSVSANTVAESHEGEFGCTLMDLRKLMELRGADAVAQISAHYGGVQEIC

AT2B1_MOUSE   TKLKTSPNEGLSGNPADLERREAVFGKNFIPPKKPKTFLQLVWEALQDVTLIILEIAAIV
AT2B2_MOUSE   RRLKTSPVEGLPGTAPDLEKRKQIFGQNFIPPKKPKTFLQLVWEALQDVTLIILEIAAII
A2ALL9_MOUSE  RRLKTSPTEGLADNTNDLEKRRQIYGQNFIPPKQPKTFLQLVWEALQDVTLIILEVAAIV
AT2B4_MOUSE   TRLKTSPIEGLSGNPADLEKRRLVFGKNVIPPKRPKTFLELVWEALQDVTLIILEIAAII

AT2B1_MOUSE   SLGLSFYQPPEGDNALCGEVSVG-EEEGEGETGWIEGAAILLSVVCVVLVTAFNDWSKEK
AT2B2_MOUSE   SLGLSFYHPPGESNEGCATAQGGAEDEGEAEAGWIEGAAILLSVICVVLVTAFNDWSKEK
A2ALL9_MOUSE  SLGLSFYAPPGEESEACGNVSGGAEDEGEAEAGWIEGAAILLSVICVVLVTAFNDWSKEK
AT2B4_MOUSE   SLVLSFYRPPGGDNEICGHIASSPEEEEEGETGWIEGAAILASVIIVVLVTAFNDWSKEK

AT2B1_MOUSE   QFRGLQSRIEQEQKFTVIRGGQVIQIPVADITVGDIAQVKYGDLLPADGILIQGNDLKID
AT2B2_MOUSE   QFRGLQSRIEQEQKFTVVRAGQVVQIPVAEIVVGDIAQIKYGDLLPADGLFIQGNDLKID
A2ALL9_MOUSE  QFRGLQSRIEQEQKFTVIRNGQLLQVPVAALVVGDIAQVKYGDLLPADGVLIQGNDLKID
AT2B4_MOUSE   QFRGLQSRIELEQKFSIIRNGQLIQLPVAEIVVGDIAQIKYGDLLPADGILIQGNDLKID

AT2B1_MOUSE   ESSLTGESDHVKKSLDKDPLLLSGTHVMEGSGRMVVTAVGVNSQTGIIFTLLGAGGEEEE
AT2B2_MOUSE   ESSLTGESDQVRKSVDKDPMLLSGTHVMEGSGRMVVTAVGVNSQTGIIFTLLGAGGEEEE
A2ALL9_MOUSE  ESSLTGESDHVRKSADKDPMLLSGTHVMEGSGRMVVTAVGVNSQTGIIFTLLGAGGEEEE
AT2B4_MOUSE   ESSLTGESDHVKKTLDKDPMLLSGTHVMEGSGRMVVTAVGVNSQTGIIFTLLGASEEEDD

AT2B1_MOUSE   KKDEKKKEKKNKKQDGAIENR--NKAKAQDGAAMEMQPLKSEEGGDGDEKDKKKANLPKK
AT2B2_MOUSE   KKDKKAKQ-----Q--------------DGAAAMEMQPLKSAEGGDAD--DKKKANMHKK
A2ALL9_MOUSE  KKDKKGKQ-----QDGAMDSSQTRAKKQDGAVAMEMQPLKSAEGGEMEEREKKKANVPKK
AT2B4_MOUSE   DDKKKKGK-----KQGAPEN--RNKAKTQDGVALEIQPLNSQEGLDSEDKEKKIARIPKK

AT2B1_MOUSE   EKSVLQGKLTKLAVQIGKAGLLMSAITVIILVLYFVIDTFWVQKRPWLAECTPIYIQYFV
AT2B2_MOUSE   EKSVLQGKLTKLAVQIGKAGLVMSAITVIILVLYFTVDTFVVNKKPWLTECTPVYVQYFV
A2ALL9_MOUSE  EKSVLQGKLTKLAVQIGKAGLVMSAITVIILVLYFVIETFVVDGRVWLAECTPVYVQYFV
AT2B4_MOUSE   EKSVLQGKLTRLAVQIGKAGLIMSVLTVVILILYFVVDNFVIQRREWLPECTPVYIQYFV

AT2B1_MOUSE   KFFIIGVTVLVVAVPEGLPLAVTISLAYSVKKMMKDNNLVRHLDACETMGNATAICSDKT
AT2B2_MOUSE   KFFIIGVTVLVVAVPEGLPLAVTISLAYSVKKMMKDNNLVRHLDACETMGNATAICSDKT
A2ALL9_MOUSE  KFFIIGVTVLVVAVPEGLPLAVTISLAYSVKKMMKDNNLVRHLDACETMGNATAICSDKT
AT2B4_MOUSE   KFFIIGVTVLVVAVPEGLPLAVTISLAYSVKKMMKDNNLVRHLDACETMGNATAICSDKT

AT2B1_MOUSE   GTLTMNRMTVVQAYINEKHYKKVPEPEAIPPNILSYLVTGISVNCAYTSKILPPEKEGGL
AT2B2_MOUSE   GTLTTNRMTVVQAYVGDVHYKEIPDPSSINAKTLELLVNAIAINSAYTTKILPPEKEGAL
A2ALL9_MOUSE  GTLTTNRMTVVQSYLGDTHYKEIPAPSALTPKILDLLVHAISINSAYTTKILPPEKEGAL
AT2B4_MOUSE   GTLTMNRMTVVQAYIGGTHYRQIPQPDVFPPKVLELIVNGISINCAYTSKIQPPEKEGGL

AT2B1_MOUSE   PRHVGNKTECALLGFLLDLKRDYQDVRNEIPEEALYKVYTFNSVRKSMSTVLKNSDGSFR
AT2B2_MOUSE   PRQVGNKTECGLLGFVLDLRQDYEPVRSQMPEEKLYKVYTFNSVRKSMSTVIKMPDESFR
A2ALL9_MOUSE  PRQVGNKTECALLGFVLDLKRDFQPVREQIPEDQLYKVYTFNSVRKSMSTVIRMPDGGFR
AT2B4_MOUSE   PRQVGNKTECGLLGFVTDLKQDYQAVRNEVPEEKLFKVYTFNSVRKSMSTVIRKPEGGFR

AT2B1_MOUSE   IFSKGASEIILKKCFKILSANGEAKVFRPRDRDDIVKTVIEPMASEGLRTICLAFRDFPA
AT2B2_MOUSE   MYSKGASEIVLKKCCKILSGAGEARVFRPRDRDEMVKKVIEPMACDGLRTICVAYRDFPS
A2ALL9_MOUSE  LFSKGASEILLKKCTNILNSNGELRGFRPRDRDDMVKKIIEPMACDGLRTICIAYRDFSA
AT2B4_MOUSE   MFSKGASEIMLRRCDRILNKEGEIKSFRSKDRDNMVRNVIEPMASEGLRTICLAYRDFDG

AT2B1_MOUSE   GEPEPEWDNENDVVTGLTCIAVVGIEDPVRPEVPEAIKKCQRAGITVRMVTGDNINTARA
AT2B2_MOUSE   -SPEPDWDNENDILNELTCICVVGIEDPVRPEVPEAIRKCQRAGITVRMVTGDNINTARA
A2ALL9_MOUSE  -IQEPDWDNENEVVGDLTCIAVVGIEDPVRPEVPEAIRKCQRAGITVRMVTGDNINTARA
AT2B4_MOUSE   --TEPSWDIEGEILTSLICIAVVGIEDPVRPEVPDAIAKCKRAGITVRMVTGDNVNTARA

AT2B1_MOUSE   IATKCGILHPGEDFLCLEGKDFNRRIRNEKGEIEQERIDKIWPKLRVLARSSPTDKHTLV
AT2B2_MOUSE   IAIKCGIIHPGEDFLCLEGKEFNRRIRNEKGEIEQERIDKIWPKLRVLARSSPTDKHTLV
A2ALL9_MOUSE  IAAKCGIIQPGEDFLCLEGKEFNRRIRNEKGEIEQERLDKVWPKLRVLARSSPTDKHTLV
AT2B4_MOUSE   IATKCGILTPKDDFLCLEGKEFNSLIRNEKGEVEQEKLDKIWPKLRVLARSSPTDKHTLV

AT2B1_MOUSE   KGIIDSTVSEQRQVVAVTGDGTNDGPALKKADVGFAMGIAGTDVAKEASDIILTDDNFTS
AT2B2_MOUSE   KGIIDSTHTEQRQVVAVTGDGTNDGPALKKADVGFAMGIAGTDVAKEASDIILTDDNFSS
A2ALL9_MOUSE  KGIIDSTTGEQRQVVAVTGDGTNDGPALKKADVGFAMGIAGTDVAKEASDIILTDDNFTS
AT2B4_MOUSE   KGIIDSTAGEQRQVVAVTGDGTNDGPALKKADVGFAMGIAGTDVAKEASDIILTDDNFTS

AT2B1_MOUSE   IVKAVMWGRNVYDSISKFLQFQLTVNVVAVIVAFTGACITQDSPLKAVQMLWVNLIMDTL
AT2B2_MOUSE   IVKAVMWGRNVYDSISKFLQFQLTVNVVAVIVAFTGACITQDSPLKAVQMLWVNLIMDTF
A2ALL9_MOUSE  IVKAVMWGRNVYDSISKFLQFQLTVNVVAVIVAFTGACITQDSPLKAVQMLWVNLIMDTF
AT2B4_MOUSE   IVKAVMWGRNVYDSISKFLQFQLTVNVVAVIVAFTGACITQDSPLKAVQMLWVNLIMDTF

AT2B1_MOUSE   ASLALATEPPTESLLLRKPYGRNKPLISRTMMKNILGHAFYQLVVVFTLLFAGEKFFDID
AT2B2_MOUSE   ASLALATEPPTETLLLRKPYGRNKPLISRTMMKNILGHAVYQLTLIFTLLFVGEKMFQID
A2ALL9_MOUSE  ASLALATEPPTESLLLRKPYGRDKPLISRTMMKNILGHAVYQLTIIFTLLFVGELFFDID
AT2B4_MOUSE   ASLALATEPPTESLLRRRPYGRNKPLISRTMMKNILGHAVYQLLIVFLLVFAGDTLFDID

AT2B1_MOUSE   SGRNAPLHAPPSEHYTIVFNTFVLMQLFNEINARKIHGERNVFEGIFNNAIFCTIVLGTF
AT2B2_MOUSE   SGRNAPLHSPPSEHYTIIFNTFVMMQLFNEINARKIHGERNVFDGIFRNPIFCTIVLGTF
A2ALL9_MOUSE  SGRNAPLHSPPSEHYTIIFNTFVMMQLFNEINARKIHGERNVFDGIFSNPIFCTIVLGTF
AT2B4_MOUSE   SGRKAPLNSPPSQHYTIVFNTFVLMQLFNEINARKIHGEKNVFAGVYRNIIFCTVVLGTF

AT2B1_MOUSE   VVQIIIVQFGGKPFSCSELSIEQWLWSIFLGMGTLLWGQLISTIPTSRLKFLKEAGHGTQ
AT2B2_MOUSE   AIQIVIVQFGGKPFSCSPLQLDQWMWCIFIGLGELVWGQVIATIPTSRLKFLKEAGRLTQ
A2ALL9_MOUSE  GIQIVIVQFGGKPFSCSPLSTEQWLWCLFVGVGELVWGQVIATIPTSQLKCLKEAGHGPG
AT2B4_MOUSE   FCQIMIVELGGKPFSCTSLTMEQWMWCLFIGIGELLWGQVISAIPTKSLKFLKEAGHGSD

AT2B1_MOUSE   KEEIPEEELAEDVEEIDHAERELRRGQILWFRGLNRIQTQIRVVNAFRSS-LYEGLEKPE
AT2B2_MOUSE   KEEIPEEELNEDVEEIDHAERELRRGQILWFRGLNRIQTQIRVVKAFRSS-LYEGLEKPE
A2ALL9_MOUSE  KDEMTDEELAEGEEEIDHAERELRRGQILWFRGLNRIQTQMEVVSTFKRSGSFQGAVRRR
AT2B4_MOUSE   KEDISRD--TEGMDEIDLAEMELRRGQILWVRGLNRIQTQIRVVKLFHNNHEVA--HKPK

AT2B1_MOUSE   SR--SSIHNFMTHPEFRIEDSEPHIPLIDDTDAEDDAPTKR-------NSSPPPSPNKNN
AT2B2_MOUSE   SR--TSIHNFMAHPEFRIEDSQPHIPLIDDTDLEEDAALKQ-------NSSPPSSLNKNN
A2ALL9_MOUSE  SSVLSQLHDVTN------LSTPTHVTL---------------------SAAKPPSAAGNP
AT2B4_MOUSE   N--RSSIHTFMTQPEYPADDELSQSFL-DIQEGNPELVSKAGTSVLLLDGEAASHDNINN

AT2B1_MOUSE   NAVDSGIHLTIEMNKSATSSSPGSPLHSLETSL
AT2B2_MOUSE   SAIDSGINLTTDTSKSATSSSPGSPIHSLETSL
A2ALL9_MOUSE  GG--ESIP-------------------------
AT2B4_MOUSE   NAVDCHQ-------VQIVASHSDSPLPSLETPV
```

Supplementary Fig. S3

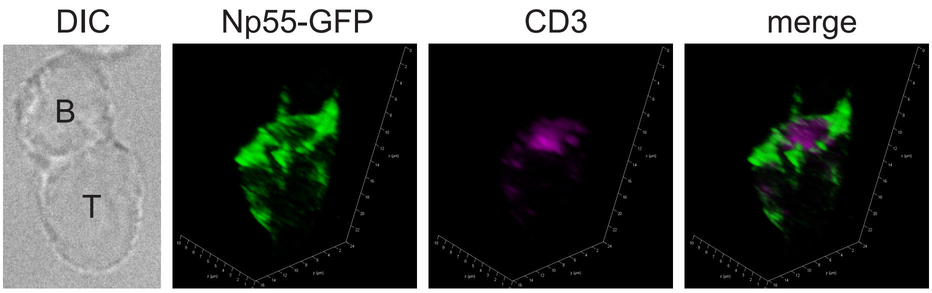
DIC
Np55-GFP
CD3
merge
B
T

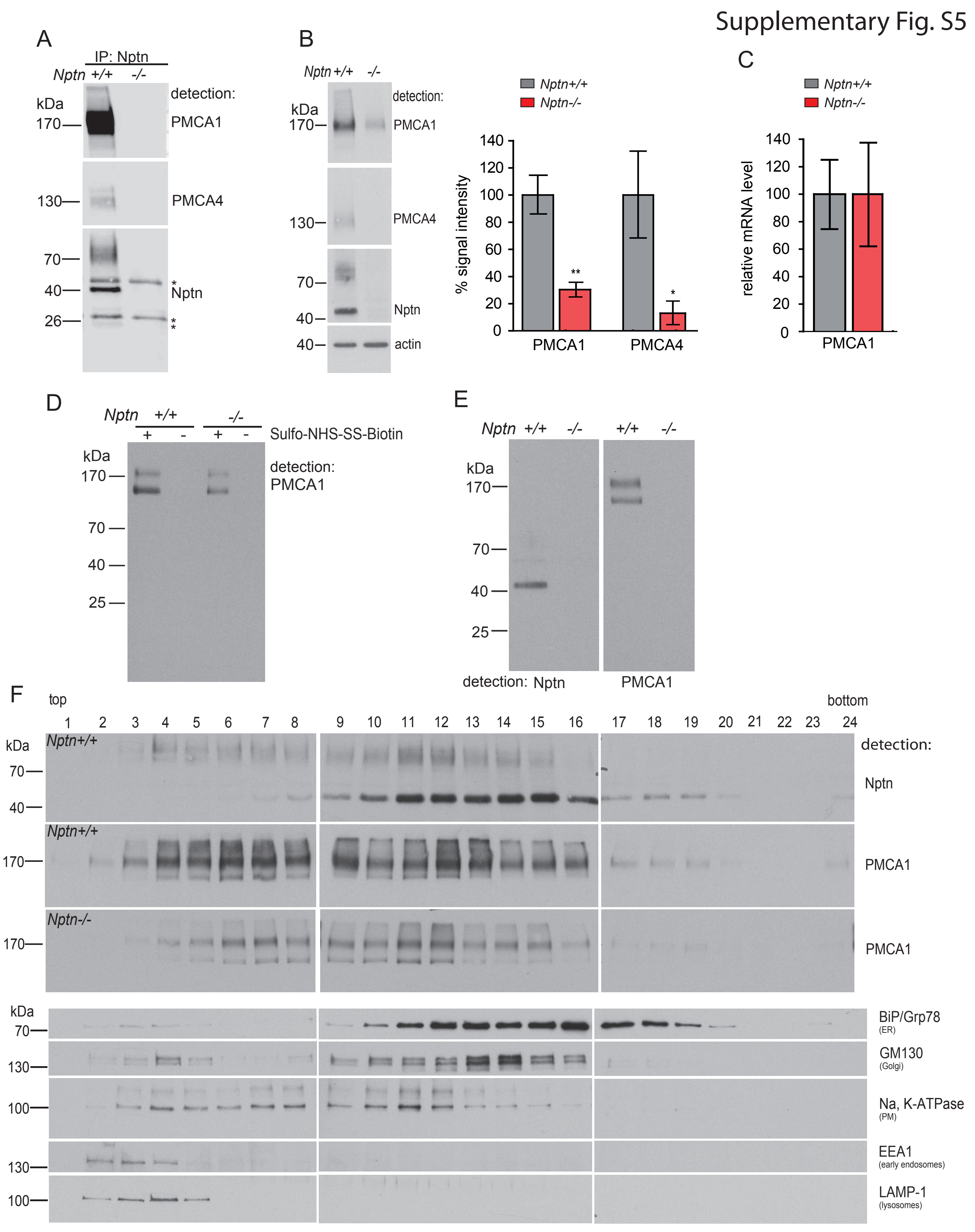
Supplementary Fig. S5
A
IP: Nptn
Nptn +/+ -/-
kDa
170
130
70
40
26
detection:
PMCA1
PMCA4
Nptn
*
*
*
B
Nptn +/+ -/-
kDa
170
130
70
40
40
detection:
PMCA1
PMCA4
Nptn
actin
Nptn+/+
Nptn-/-
% signal intensity
0
20
40
60
80
100
120
140
**
*
PMCA1
PMCA4
C
Nptn+/+
Nptn-/-
relative mRNA level
0
20
40
60
80
100
120
140
PMCA1
D
Nptn +/+ -/-
+ - + -
Sulfo-NHS-SS-Biotin
kDa
170
70
40
25
detection:
PMCA1
E
Nptn +/+ -/- +/+ -/-
kDa
170
70
40
25
detection: Nptn
PMCA1
F
top
bottom
1 2 3 4 5 6 7 8 9 10 11 12 13 14 15 16 17 18 19 20 21 22 23 24
kDa
70
40
170
170
Nptn+/+
Nptn+/+
Nptn-/-
detection:
Nptn
PMCA1
PMCA1
kDa
70
130
100
130
100
BiP/Grp78
(ER)
GM130
(Golgi)
Na, K-ATPase
(PM)
EEA1
(early endosomes)
LAMP-1
(lysosomes)

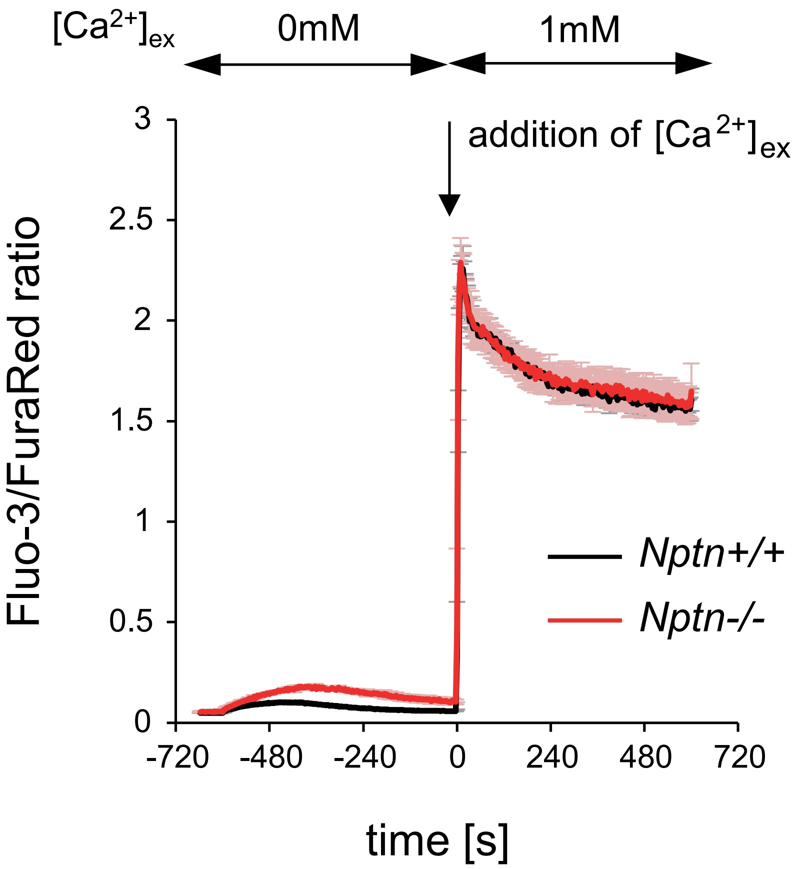

$[Ca^{2+}]_{ex}$
0mM
1mM
addition of $[Ca^{2+}]_{ex}$
Fluo-3/FuraRed ratio
3
2.5
2
1.5
1
0.5
0
-720
-480
-240
0
240
480
720
time [s]
Nptn+/+
Nptn-/-

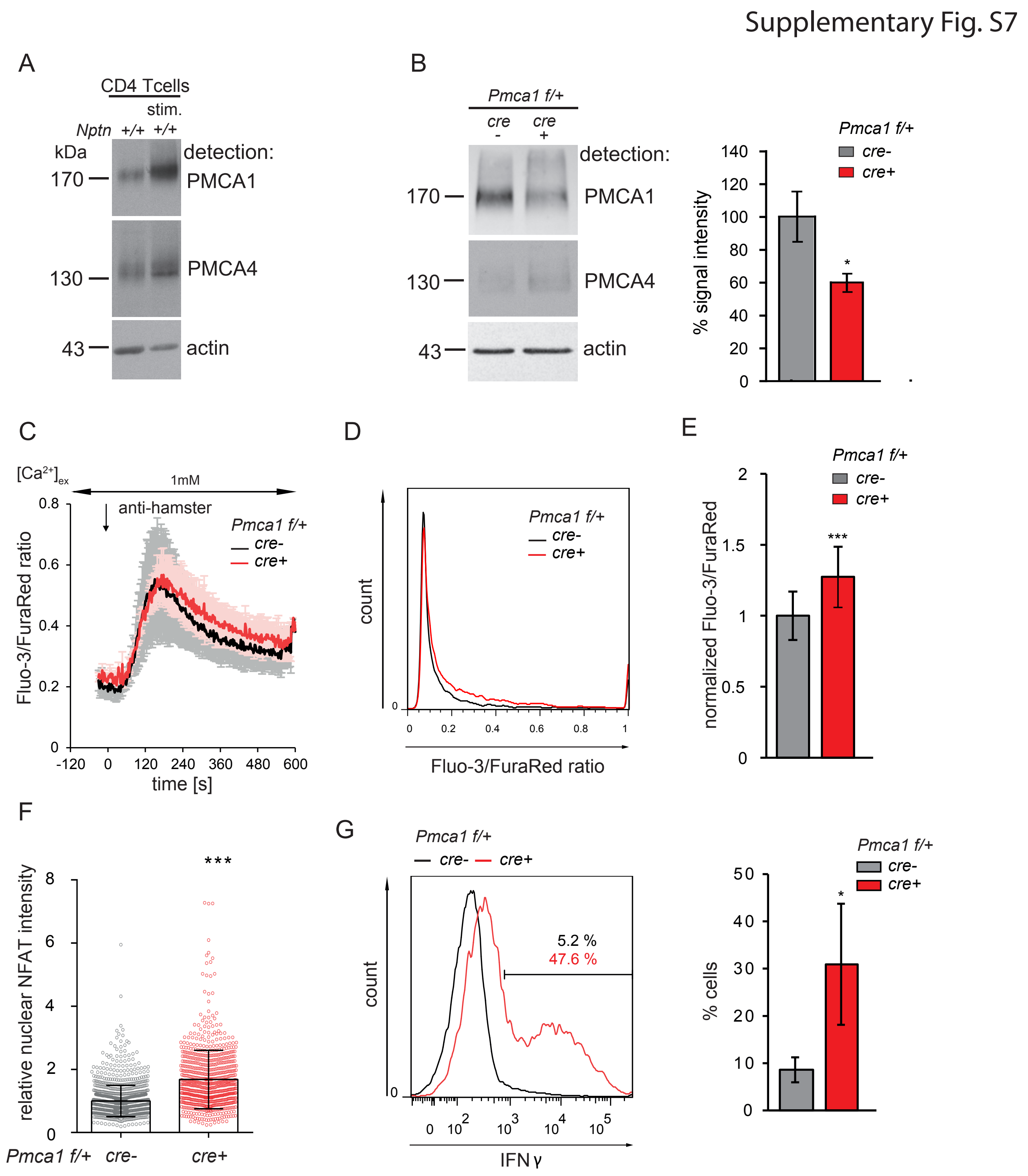

Supplementary Fig. S7
A
CD4 Tcells
stim.
Nptn +/+ +/+
kDa
detection:
170
PMCA1
130
PMCA4
43
actin
B
Pmca1 f/+
cre -
cre +
detection:
170
PMCA1
130
PMCA4
43
actin
Pmca1 f/+
cre-
cre+
% signal intensity
140
120
100
80
60
40
20
0
*
C
[Ca2+]ex
1mM
anti-hamster
Pmca1 f/+
cre-
cre+
Fluo-3/FuraRed ratio
0.8
0.6
0.4
0.2
0
-120 0 120 240 360 480 600
time [s]
D
Pmca1 f/+
cre-
cre+
count
0
0 0.2 0.4 0.6 0.8 1
Fluo-3/FuraRed ratio
E
Pmca1 f/+
cre-
cre+
normalized Fluo-3/FuraRed
2
1.5
1
0.5
0
***
F
***
relative nuclear NFAT intensity
8
6
4
2
0
Pmca1 f/+ cre- cre+
G
Pmca1 f/+
cre- cre+
5.2 %
47.6 %
count
0
0 10^2 10^3 10^4 10^5
IFN γ
Pmca1 f/+
cre-
cre+
% cells
50
40
30
20
10
0
*

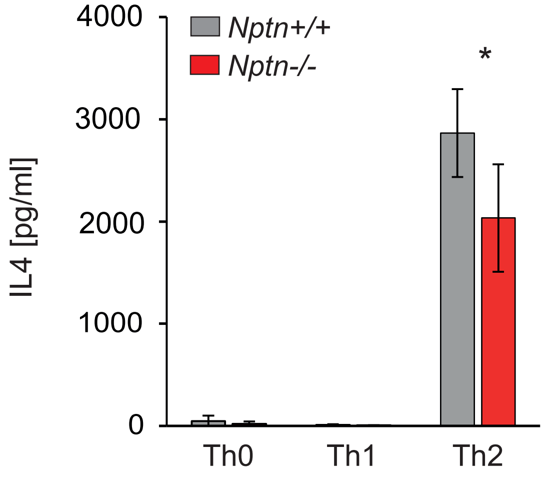

4000
3000
2000
1000
0
IL4 [pg/ml]
Nptn+/+
Nptn-/-
*
Th0
Th1
Th2

Supplementary Table S1: Quantification of cytosolic Ca^{2+} dynamics in *Nptn*$^{-/-}$ T cells

Mean $[Ca^{2+}]_i$ baseline and peak levels upon TCR stimulation (Fig. 2A)

	Nptn$^{+/+}$	***Nptn***$^{-/-}$	**p**
baseline [a.u.]	0.125 ± 0.014	0.213 ± 0.033	**0.004**
Peak [a.u.]	0.410 ± 0.074	0.399 ± 0.045	0.86
time to peak [s]	124.8 ± 9.7	121.3 ± 20.4	0.79

Exponential fit of $[Ca^{2+}]_i$ clearance after TCR-induced Ca^{2+} influx (Fig. 2A)

	Nptn$^{+/+}$	***Nptn***$^{-/-}$	
Y0 [a.u.]	0.428 ± 0.007	0.417 ± 0.009	n.a.
Plateau [a.u.]	0.115 ± 0.002	0.206 ± 0.004	
K [1/s]	0.0092 ± 0.0003	0.0078 ± 0.0006	
$T_{1/2}$ [s]	75.3	88.4	
initial rate [a.u./s]	-0.0029	-0.0016	
R^2	0.9857	0.9402	

Exponential fit of $[Ca^{2+}]_i$ clearance after SOCE induced by SERCA inhibition (Fig. 5E)

	Nptn$^{+/+}$	***Nptn***$^{-/-}$	
Y0 [a.u.]	2.079 ± 0.042	2.104 ± 0.064	n.a.
Plateau [a.u.]	0.603 ± 0.016	0.835 ± 0.026	
K [1/s]	0.354 ± 0.026	0.306 ± 0.038	
$T_{1/2}$ [s]	1.96	2.27	
initial rate [a.u./s]	-0.522	-0.388	
R^2	0.9929	0.9784	

a.u., arbitrary units derived from the Fluo-3/FuraRed intensity ratio

Supplementary Table S2: Quantification of cytosolic Ca^{2+} dynamics in *Pmca1*$^{f/+}$ T cells

Mean $[Ca^{2+}]_i$ baseline and peak levels upon TCR stimulation (Suppl. Fig. S7)

	Cre$^-$	*Cre*$^+$	p
baseline [a.u.]	0.177 ± 0.037	0.226 ± 0.052	**0.0021**
Peak [a.u.]	0.577 ± 0.166	0.533 ± 0.088	0.420
time to peak [s]	164.7 ± 8.1	170.0 ± 8.0	0.613

Exponential fit of $[Ca^{2+}]_i$ clearance after TCR-induced Ca^{2+} influx (Suppl. Fig. S7)

	Cre$^-$	*Cre*$^+$	
Y0 [a.u.]	0.629 ± 0.009	0.575 ± 0.008	n.a.
Plateau [a.u.]	0.303 ± 0.003	0.322 ± 0.007	
K [1/s]	0.0086 ± 0.0004	0.0060 ± 0.0005	
$T_{1/2}$ [s]	80.8	115	
initial rate [a.u./s]	-0.0028	-0.0015	
R^2	0.9767	0.9505	

Exponential fit of $[Ca^{2+}]_i$ clearance after SOCE induced by SERCA inhibition (Fig. 5H)

	Cre$^-$	*Cre*$^+$	
Y0 [a.u.]	2.200 ± 0.064	2.278 ± 0.046	n.a.
Plateau [a.u.]	0.866 ± 0.024	1.062 ± 0.018	
K [1/s]	0.382 ± 0.048	0.344 ± 0.033	
$T_{1/2}$ [s]	1.82	2.01	
initial rate [a.u./s]	-0.509	-0.418	
R^2	0.9802	0.9878	

a.u., arbitrary units derived from the Fluo-3/FuraRed intensity ratio
